# Habitat‐Specific Design Strategies for Untethered Soft Robots

**DOI:** 10.1002/advs.78287

**Published:** 2026-10-18

**Authors:** Siyang Li, Zhikun Miao, Tuck‐Whye Wong, Tiefeng Li

**Affiliations:** ^1^ Center for X‐Mechanics Department of Engineering Mechanics Zhejiang University Hangzhou China; ^2^ Sustainable and Smart Materials Laboratory Department of Biomedical Engineering and Health Sciences Universiti Teknologi Malaysia Johor Bahru Malaysia

**Keywords:** future of robotics, legged robot, medical robotics, robot, robot design, robot motion, soft robotics, underwater robot

## Abstract

Untethered soft robots are governed by habitat‐specific constraints because operation without physical tethers requires their compliant structures, actuation mechanisms, and energy‐delivery pathways to remain compatible with the environment. Adopting a habitat‐centric framework, this review compares untethered soft robots across terrestrial, aquatic, aerial, and in vivo environments. On terrestrial substrates, design strategies are shaped by locomotion‐specific demands, including contact modulation in crawling, energy storage and release in jumping, switchable adhesion in climbing, and granular fluidization in burrowing. In aquatic habitats, the dominant constraint shifts with depth, from capillary support at the air‐water interface, to hydrodynamic drag in shallow water, and to hydrostatic pressure in the deep sea. In aerial environments, strict mass and power limits favor actively flapping insect‐scale robots, buoyancy‐assisted vehicles, and passively dispersed aeroelastic structures. In vivo, miniaturization and biocompatibility confine the viable design space to magnetic, acoustic, and biohybrid actuation. A multi‐environment comparison shows that no single actuation modality is universally optimal. Each habitat filters actuation strategies by the compatibility between energy transmission and the local medium. Material longevity, onboard energy autonomy, multi‐environment transition, extraterrestrial operation, and perception and feedback control are identified as cross‐cutting challenges whose resolution is required to translate laboratory demonstrations into real‐world deployment.

## Graphical abstract

Habitat shapes the design of untethered soft robots. Across land, water, air, and living tissues, environmental constraints govern viable combinations of compliant structures, actuation mechanisms, and energy delivery. This review connects these constraints with robotic performance and identifies design principles for advancing adaptable, durable, and controllable systems beyond laboratory demonstrations.

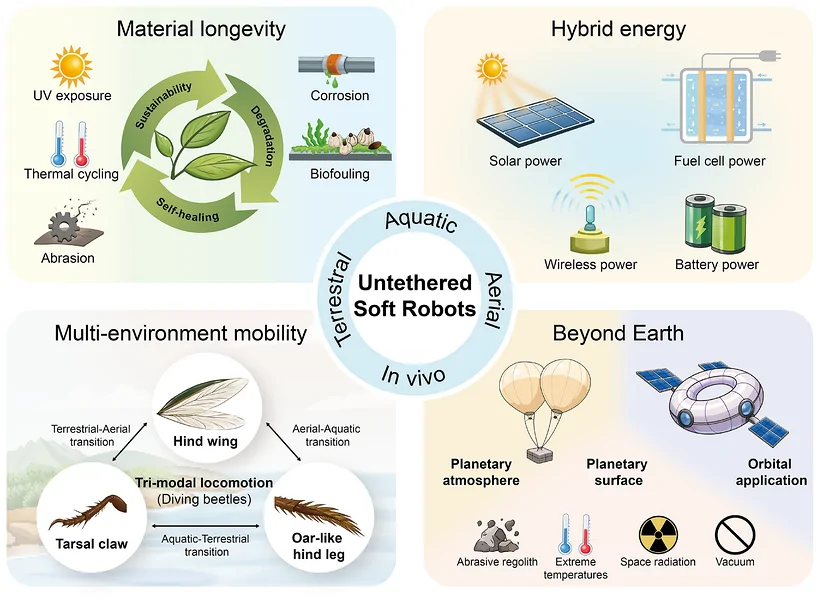

## 1 Introduction

Untethered soft robots are increasingly being developed for operation in environments that remain challenging for conventional rigid robotic systems, including complex terrestrial terrains, aquatic environments spanning from the water surface to the deep sea, aerial domains with severe mass constraints, and confined in vivo spaces within living organisms [1, 2, 3, 4, 5, 6]. In these environments, successful operation depends not only on locomotion performance, but also on the ability to actuate, manage power, and remain functional under habitat‐specific physical, chemical, and biological constraints [2, 7].

Unlike conventional robots, whose rigid housings mechanically decouple internal components from the surrounding environment, soft robots remain in continuous mechanical coupling with their operating medium through deformable bodies and compliant interfaces [8, 9, 10, 11]. Environmental conditions therefore act directly on locomotion efficiency, force generation, energy consumption, and structural durability [1]. The design requirements for a robot moving across granular terrain [12, 13] differ fundamentally from those for a robot swimming under hydrodynamic drag [14, 15], flying under strict lift‐to‐weight constraints [6, 16], or navigating through biological tissues [17, 18]. Consequently, materials, structures, and control strategies that perform effectively in one habitat may become ineffective or even infeasible in another.

This habitat specificity is most consequential for untethered systems. The coupling between robot design and habitat becomes particularly consequential when physical tethers are removed. Energy, actuation, sensing, and control must then be supplied through combinations of onboard resources, non‐contact external inputs, and environmental resources [2, 3]. The feasibility of these combinations depends on the operating medium, making habitat constraints central to system integration.

For this review, an untethered soft robot performs the reported task without a physical connection to external equipment, with compliant deformation contributing substantively to propulsion, shape adaptation, load support, or manipulation. Hybrid rigid–soft systems are included when their compliant components provide these functions. Physical untethering does not require onboard energy storage, computation, or autonomous decision‐making: magnetic, acoustic, optical, and radiofrequency power delivery or field actuation, wireless commands, external non‐contact sensing, and offboard computation are permitted. Eligibility is assessed for the demonstrated configuration and task, rather than for a platform or publication as a whole. Tethered configurations, including connections used only for sensing or actuation, and component‐level demonstrations serve as background or enabling studies; their performance does not establish that of an untethered system. Comparisons of untethered performance are restricted to configurations meeting these criteria.

Existing reviews have summarized soft robotics from perspectives such as materials [19, 20], actuation mechanisms [7, 21], fabrication techniques [22], bioinspired design [23], and application‐specific developments [4, 5, 24]. While these classifications provide valuable insights into individual technological aspects, they often overlook the role of the operating environment as the upstream constraint governing design decisions. For untethered soft robots, habitat frequently determines which materials, structures, actuation strategies, and energy‐delivery approaches are feasible before any other design decision is made. A habitat‐centric perspective therefore provides an alternative framework for understanding the evolution of untethered soft robotic systems and identifying common design principles across seemingly unrelated platforms.

In this review, we organize untethered soft robots according to four major operational habitats: terrestrial, aquatic, aerial, and in vivo. For each habitat, we identify the dominant environmental constraints and discuss how these constraints shape locomotion strategies, structural architectures, and actuation mechanisms. We further compare major actuation modalities across habitats and examine how energy transmission pathways and environmental interactions determine their applicability. Finally, we discuss future challenges and opportunities, including material longevity, onboard energy autonomy, multi‐environment operation, extraterrestrial operation, and perception and feedback control. Through this habitat‐centric framework, we aim to establish a unified understanding of how environmental constraints govern the design and functionality of untethered soft robots across diverse operational domains.

## 2 Habitat‐Specific Design Strategies

We organize the review using habitat constraints as the common starting point and the corresponding design responses as the secondary level. On land, the relevant distinction is how a robot interacts with the substrate; in water, the discussion follows the transition from interface forces to submerged propulsion and pressure‐tolerant operation. In air, systems are grouped by weight support and flight regime. For in vivo applications, the discussion follows the functional sequence from wireless actuation to navigation and cargo delivery. These secondary groupings reflect different combinations of physical constraints rather than an identical taxonomy imposed on all four habitats.

The criteria below are proposed reporting measures rather than universal numerical pass–fail thresholds: acceptable values depend on the mission, robot scale, and test conditions, and reported performance values should be interpreted within that context.

### 2.1 Terrestrial Soft Robots

Terrestrial environments expose soft robots to irregular, deformable, and largely unpredictable conditions. Artificial terrains such as stairs combine ramps with discrete height discontinuities, requiring stable body support and accurate foothold placement to avoid tumbling [25, 26, 27]. Natural terrains are more challenging: rocky ground creates uneven contact heights; granular substrates yield and reorganize under load, often causing slip‐stick transitions governed by friction and compaction [12, 28, 29, 30]; and dense vegetation introduces lateral deflection, entanglement, and geometric confinement [31]. Across these settings, the central design requirement is consistent. Terrestrial soft robots must combine sufficient compliance to adapt to irregular surfaces with sufficient force and structural support to overcome gravity and frictional resistance on diverse substrates.

Wheeled systems provide high speed and payload capacity on firm, flat ground, but their performance degrades rapidly when obstacles become comparable to wheel size or when the workspace is cluttered or spatially constrained. Under such conditions, locomotion depends increasingly on distributed compliance, multi‐point contact, and reconfigurable body or limb kinematics to maintain traction and stability [32, 33]. Soft robots are well suited to these conditions because deformable structures can redistribute contact forces, absorb impact, and adapt their geometry to terrain variation [34, 35, 36]. Because these constraints do not vary along a single continuous environmental axis, the dominant mechanical challenge is more naturally distinguished by the form of robot–substrate interaction. Accordingly, successful terrestrial operation is defined here as stable and controllable locomotion over the intended substrate while maintaining useful locomotion speed, terrain‐negotiation capability, payload capacity, and endurance.

Successful terrestrial operation requires completion of the intended route or maneuver without persistent slipping, stalling, or loss of support. Evaluation should report absolute and body‐length‐normalized speed, payload‐to‐body‐mass ratio, and operating duration, together with substrate properties and task completion across repeated trials. Additional measures depend on the locomotion mode: obstacle dimensions and landing accuracy for jumping, slope and attachment reliability for climbing, and penetration depth and directional error for burrowing. These conditions distinguish a demonstrated task capability from speed measured on an uncomplicated test surface.

On relatively flat terrain without large obstacles, locomotion is primarily governed by frictional interactions between the robot and the substrate (Figure 1A). The central challenge is therefore not obstacle negotiation, but the generation and modulation of anisotropic friction to convert cyclic body deformation into net directional motion. Two broad design strategies are commonly used. The first is body‐driven crawling, in which deformation of the main structure simultaneously generates propulsion and modulates contact conditions. Within this category, two‐anchor locomotion is especially effective because alternating anchoring and release can transform cyclic deformation into net forward motion [37, 38]. Liang et al. demonstrated this strategy at small scale using a curved piezoelectric unimorph, whose resonant expansion and contraction alternately engaged anterior and posterior contacts with the substrate, enabling rapid forward locomotion at small scales (Figure 1B) [39]. The second strategy is leg‐driven crawling, in which independently actuated appendages provide greater dexterity over foothold placement and gait generation. Compared with body‐driven systems, leg‐driven architectures generally offer improved maneuverability and obstacle‐negotiation capability, albeit at the cost of increased mechanical and control complexity. Tolley et al. developed a pneumatically actuated quadruped, whose independently controlled limbs enabled diverse gait patterns and slope traversal when sufficient frictional counterforces were available to prevent slippage [40]. At smaller scales, Wang et al. exploited light‐responsive anisotropic friction by integrating photothermal graphene‐PDMS micropillar arrays on the robot, enabling directional locomotion across substrates with different roughness, wetness, and inclination angles (Figure 1C) [41]. Overall, crawling robots highlight the central role of friction management in terrestrial locomotion. While body‐driven architectures favor structural simplicity and robustness, leg‐driven systems provide greater locomotion versatility and environmental adaptability. The choice between these approaches is therefore largely dictated by the required balance between mechanical complexity, maneuverability, and terrain negotiability.

**FIGURE 1:**
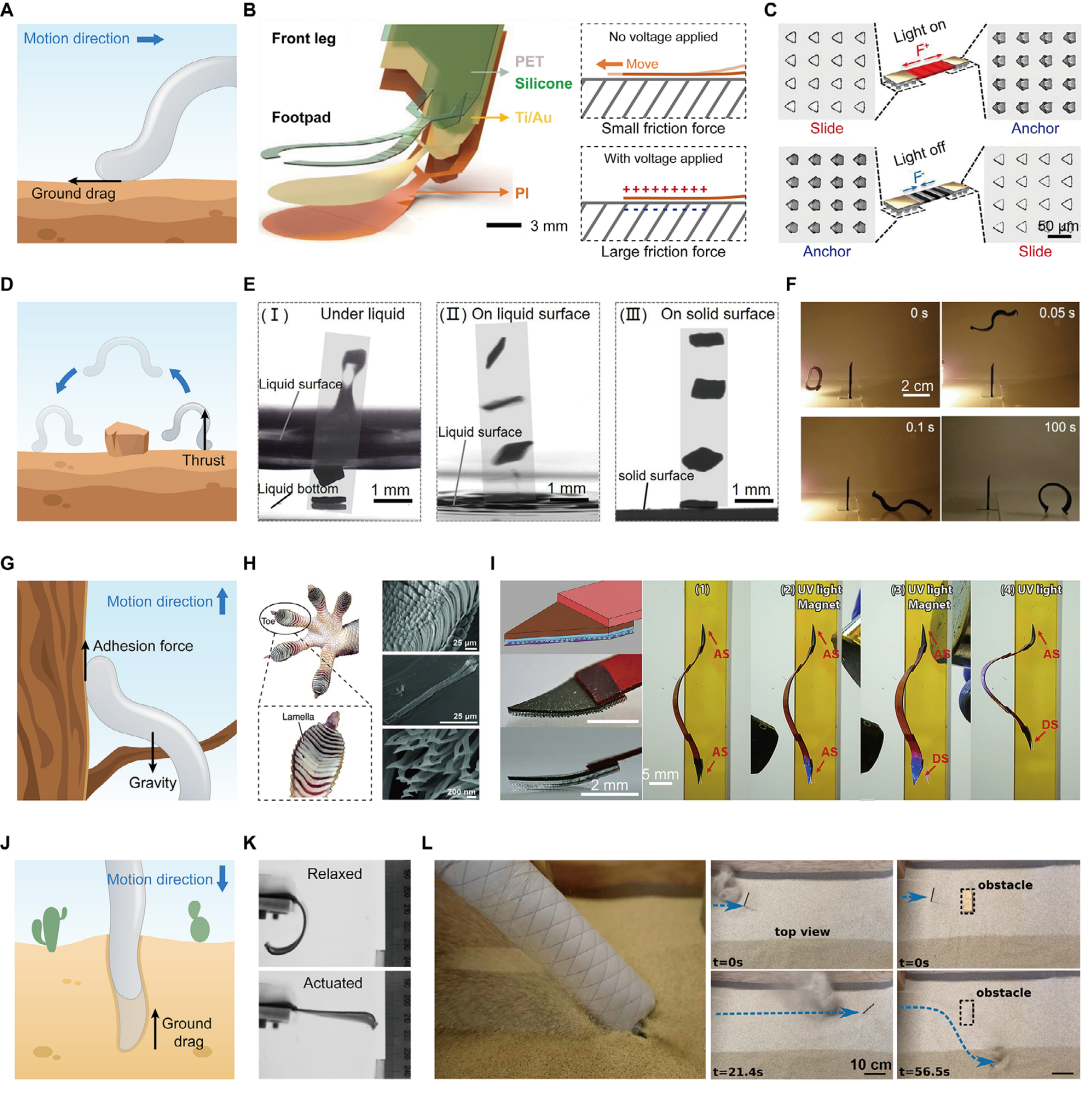
Terrestrial soft robotic systems and enabling mechanisms organized by substrate interaction and locomotion mode. The dominant design constraint shifts from contact modulation in flat‐ground crawling, to impulsive energy release in jumping, to switchable adhesion in climbing, and to drag reduction in granular media. (A) Schematic of friction‐dominated locomotion on flat ground. (B) Body‐driven crawling using a piezoelectrically resonant unimorph with electrostatic footpads to alternate anterior‐posterior anchoring. Reproduced with permission [39]. Copyright 2021, American Association for the Advancement of Science. (C) Gait sequence of the light‐driven Geca‐Robot, exploiting photothermally switched anisotropic friction across varying substrates. Reproduced with permission [41]. Copyright 2020, Elsevier Ltd. (D) Schematic of jumping locomotion for obstacle crossing. (E) High‐speed images of cavitation‐driven jumping launches across three diverse environments: underwater (I), at liquid surfaces (II), and on wet solid substrates (III). Reproduced with permission [44]. Copyright 2025, American Association for the Advancement of Science. (F) Snap‐through jumping of a liquid crystal elastomer (LCE)‐carbon nanotube (CNT) composite that converts gradual photothermal curling into sudden elastic release.Reproduced with permission [46]. Copyright 2019, Wiley‐VCH GmbH. (G) Schematic of climbing on a vertical surface, showing upward motion, the attachment force supporting the robot, and the downward direction of gravity. (H) Hierarchical fibrillar structures on the gecko toe.Reproduced with permission [51, 52]. Copyright 2006, The National Academy of Sciences of the USA, and Copyright 2012, WILEY‐VCH Verlag GmbH & Co. KGaA, Weinheim. (I) Gecko‐inspired mushroom‐tipped micropillar arrays integrated with an LCE trunk for photo‐driven climbing on vertical walls. Reproduced with permission [53]. Copyright 2023, Elsevier Inc. (J) Schematic of movement through granular media. (K) SMA‐actuated curling limbs whose residual C‐shaped curvature distributes load across yielding substrates without continuous gait adaptation, shown in relaxed (top) and activated (bottom) states. Reproduced with permission [54]. Copyright 2018, American Association for the Advancement of Science. (L) Tip‐extending burrowing robot with granular fluidization for steered subsurface navigation, including controlled horizontal burrowing and obstacle avoidance through directional turning. Reproduced with permission [13]. Copyright 2021, American Association for the Advancement of Science.

As terrain complexity increases and obstacle height becomes comparable to or exceeds the robot's characteristic body dimension, friction‐based crawling alone becomes insufficient for efficient locomotion. Under such conditions, jumping offers an effective strategy for rapidly traversing obstacles, discontinuous terrain, and confined spaces that would otherwise require complex path planning or prolonged climbing maneuvers (Figure 1D). The dominant design challenge shifts from friction management to the storage and rapid release of mechanical energy. Successful jumping therefore depends on maximizing energy density while maintaining structural durability, directional control, and landing stability. An obstacle‐crossing jump comprises preparation, impulsive take‐off, and an airborne phase followed by landing. Before take‐off, posture adjustment and energy storage establish the conditions for release; the resulting impulse sets the launch velocity and direction. For an approximately ballistic aerial phase, these initial conditions determine whether the body clears the obstacle and reaches the intended landing region. Directional control in the examples below is therefore mainly exerted before or during take‐off, rather than through demonstrated steering in mid‐air.

One approach uses combustion and phase‐transition actuators to generate large impulsive forces within milliseconds. Tolley et al. used three pneumatic limbs to set the pre‐launch orientation of a combustion‐powered soft hopper [42]. Selective inflation tilted the body toward the intended direction, after which rapid expansion of the central combustion actuator supplied the take‐off impulse. This separation of posture setting and energy release enabled directed jumps and targeted landing on an elevated object. While combustion enables high instantaneous power output, repeated impulsive loading can induce mechanical failures in the electronics and fittings. To address this issue, Bartlett et al. introduced a graded‐stiffness architecture fabricated by multi‐material 3D printing, in which a rigid core transitioned gradually to a compliant exterior, reducing stress concentration and improving durability under repeated jumping cycles [43]. Wang et al. used laser‐induced cavitation to generate impulsive launches in different surrounding media (Figure 1E) [44]. Changing the irradiation position shifted the point of force application: off‐center forcing produced rotation and an oblique launch, allowing the take‐off direction and horizontal displacement to be adjusted.

An alternative strategy relies on the accumulation and sudden release of elastic strain energy through structural instability. Such approaches are particularly attractive for small‐scale robots where onboard volume and power availability are limited. Arazoe et al. demonstrated a humidity‐responsive carbon nitride polymer film that gradually bent during moisture absorption and rapidly released stored elastic energy upon dehydration, producing vertical jumping motion [45]. Ahn et al. used a light‐responsive LCE–CNT strip with magnetic end connections to form a closed loop that stored elastic energy under irradiation (Figure 1F) [46]. Release of the magnetic connection allowed rapid deformation and take‐off; the irradiation angle controlled the pre‐release deformation and jumping trajectory. The robot crossed walls or reached elevated steps, with reported jumping distances of approximately eight body lengths and heights of five body heights. These examples show that obstacle clearance depends on both the available take‐off impulse and its direction, while repeatable operation additionally requires structural recovery and reliable landing.

Vertical and near‐vertical surfaces impose a fundamentally different locomotion challenge from horizontal terrain. In these environments, gravity acts continuously as a detachment force, promoting sliding and loss of contact throughout the locomotion cycle (Figure 1G). Consequently, the dominant design requirement shifts from propulsion generation to the establishment of reliable, reversible adhesion. Effective climbing robots must generate sufficient attachment force to support body weight while simultaneously enabling rapid and repeatable detachment to sustain locomotion [47, 48]. Several active adhesion strategies have been explored to address this challenge. Vacuum‐based adhesion generates attachment through pressure differentials between the contact interface and the surrounding environment. Tang et al. demonstrated a soft suction system employing a spiral pneumatic channel that deformed into a dome‐shaped suction cup upon inflation [49]. Alternatively, electroadhesion utilizes electrostatic attraction between charged electrodes and the contact surface. Gu et al. developed a polyimide‐electrode sandwich structure capable of generating reversible adhesion under applied voltage [50]. Although these approaches can provide substantial attachment forces, they generally require continuous energy input to maintain adhesion, reducing their suitability for energy‐limited untethered systems. To improve energy efficiency, researchers have increasingly adopted passive or mechanical switchable mechanisms inspired by dry adhesive interfaces. Hierarchical fibrillar surfaces generate attachment through van der Waals interactions and can be engaged or released through controlled loading and peeling motions (Figure 1H) [51, 52]. Because attachment can be maintained without continuous energy consumption, such systems are particularly attractive for untethered climbing robots. Sun et al. implemented this strategy by integrating mushroom‐tipped micropillar adhesive arrays with a liquid crystal elastomer (LCE) trunk, enabling photo‐driven climbing and detachment on vertical surfaces (Figure 1I) [53]. These examples show that the design of climbing soft robots is governed by the balance between attachment strength, release efficiency, and energy consumption. For untethered systems, adhesion strategies that minimize continuous power demand while maintaining reliable load support are generally more advantageous than continuously powered attachment mechanisms.

Granular substrates require a distinction between locomotion on the surface and burrowing below it (Figure 1J). At the surface, yielding and rearrangement of grains alter support and traction, making limb geometry important for limiting sinkage [12]. Huang et al. developed untethered robots with naturally curved SMA–elastomer limbs that supported the body and its onboard components (Figure 1K) [54]. Electrical heating actuated the SMA and changed limb curvature; cooling allowed elastic recovery. Repeated limb deformation changed the body–substrate contact configuration and generated locomotion on loose granular surfaces. Mechanically, net advance requires these contact forces to produce different body displacements during the actuation and recovery portions of the cycle; cyclic contraction alone is not a complete description of propulsion. The curved compliant limbs provide load support while accommodating uneven contacts, but this demonstration should not be interpreted as subsurface burrowing.

Below the surface, the robot must displace surrounding grains under confinement, while spatial variations in resistance can deflect its trajectory [55, 56, 57]. Naclerio et al. addressed this regime with a tip‐extending robot that supplied air through a nozzle at the advancing tip to fluidize the nearby grains and reduce penetration resistance (Figure 1L) [13]. Directional control of the interaction at the tip enabled steering around buried obstacles. This example illustrates a resistance‐reduction strategy for burrowing, distinct from the load‐support and traction requirements of the SMA‐legged surface robot.

Terrestrial control must connect information about contact and body motion to changes in gait, adhesion, or launch posture. Pressure and strain measurements can reveal contact loss or excessive deformation, while vision can provide route and obstacle information. Liang et al. demonstrated an untethered configuration with a battery, control circuit, and photoresistors that followed a designated 27.9 cm S‐shaped light‐defined path in 36.9 s [39]. This establishes sensor‐informed path following under the reported conditions. It should be distinguished from the faster tethered demonstrations in the same study and from autonomous navigation through previously unknown terrain.

To date, terrestrial soft robots illustrate how locomotion strategies evolve in response to increasing environmental complexity. As the dominant challenge shifts from friction management on relatively flat surfaces to obstacle negotiation, vertical adhesion, and movement through highly resistive granular media, the corresponding robotic architectures, actuation mechanisms, and structural designs must adapt accordingly. These examples highlight the strong coupling between environmental constraints and robotic functionality, a relationship that becomes even more pronounced in aquatic environments where the surrounding medium itself governs locomotion performance.

### 2.2 Aquatic Soft Robots

Aquatic habitats cover more than 70% of the Earth's surface and encompass a wide range of physical conditions that vary substantially with depth. Unlike terrestrial habitats, where locomotion is governed primarily by contact mechanics and gravity, aquatic locomotion is dominated by interactions with the surrounding fluid medium. Water is approximately 50 times more viscous and 800 times denser than air, so aquatic locomotion is subject to much greater hydrodynamic resistance [58, 59]. In addition, aquatic soft robots must maintain waterproof integrity, regulate buoyancy, withstand fluid disturbances, and tolerate progressively increasing hydrostatic pressure. Consequently, the dominant environmental constraint is not constant throughout the water column, but changes systematically with depth. Aquatic examples are organized from surface operation to shallow‐water swimming and deep‐sea deployment. This sequence highlights changes in the relative importance of capillary support, hydrodynamic interaction, and pressure tolerance; it does not imply that hydrodynamic forces disappear at depth or that every surface robot relies exclusively on capillarity.

Successful aquatic operation requires completing the intended trajectory or task while preserving structural and functional integrity. Relevant measures include speed, turning radius or heading error, depth‐control error, operating duration, and energy consumption under specified flow, temperature, and depth conditions. Pressure tolerance should identify whether the robot remained operational under load or merely survived exposure. For surface robots, interface stability and wetting state are additional requirements; for submerged systems, the demonstrated depth and resistance to flow disturbances define the operating conditions against which performance should be assessed.

Near the air‐water interface, surface tension governs the locomotion of millimeter‐scale organisms and robots, supporting their weight only below a critical size threshold (Figure 2A) [60, 61, 62, 63]. Across all depth regimes, currents, waves, and turbulence introduce dynamic disturbances that demand adaptive locomotion strategies (Figure 2B). Hydrostatic pressure increases linearly with depth (Figure 2C), reaching 110 MPa in the Mariana Trench [64, 65]. At such depths, pressure can compress structural components, stiffen polymeric materials toward the glass transition, and damage onboard electronics [66, 67, 68, 69].

**FIGURE 2:**
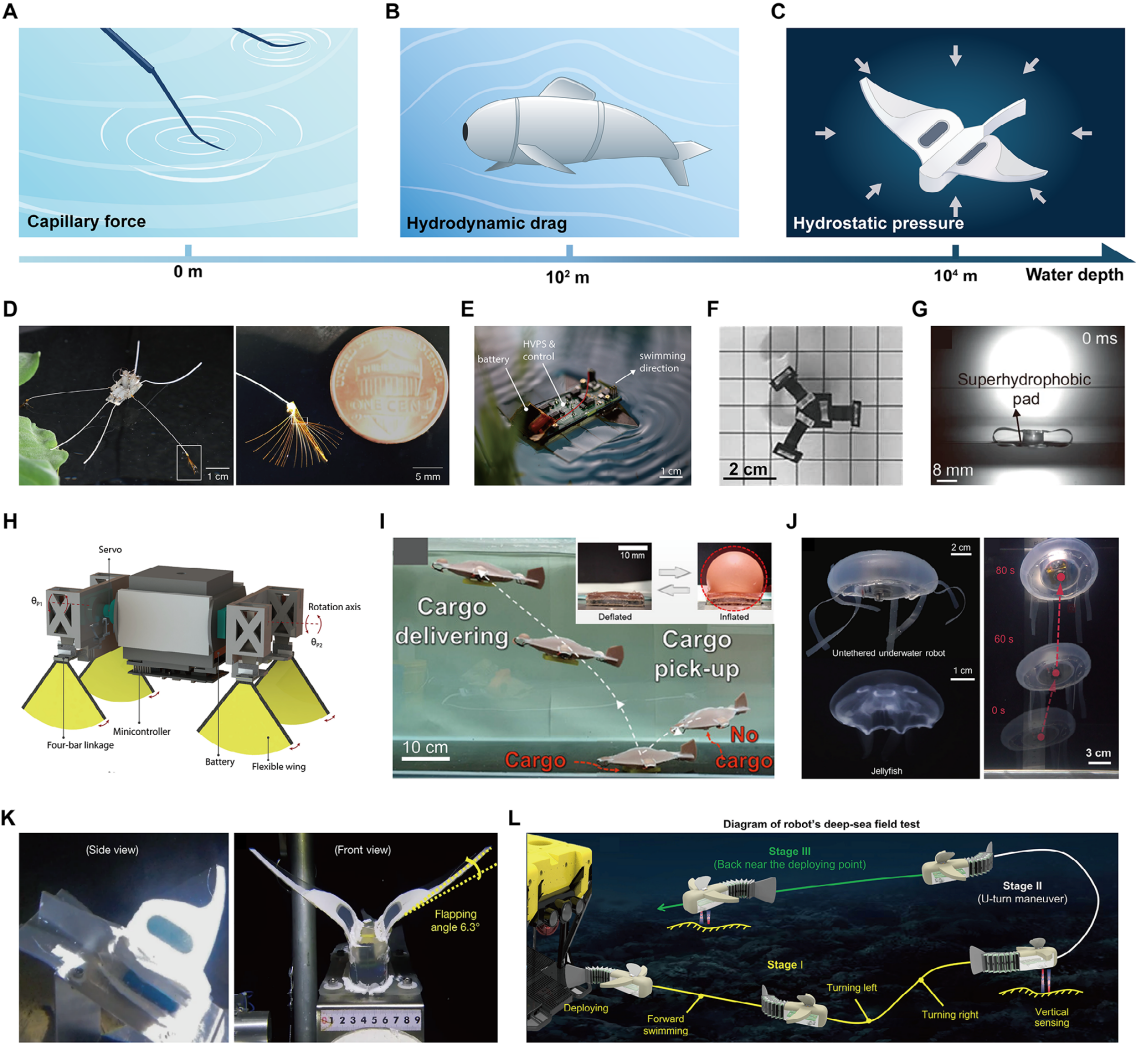
Aquatic soft robotic systems and enabling mechanisms organized from surface operation to shallow‐water swimming and deep‐sea deployment. (A) Capillary support at the interface, where mass distribution and wetting state determine viability. (B) Hydrodynamic drag dominance in shallow water, requiring effective thrust generation and three‐dimensional maneuverability. (C) Hydrostatic pressure dominance in the deep sea, requiring pressure‐tolerant structures. (D) A semiaquatic robot with self‐morphing fans at the ends of its middle legs, exploiting elastocapillary deployment for surface propulsion. Reproduced with permission [76]. Copyright 2025, American Association for the Advancement of Science. (E) An untethered, autonomous water‐walking robot propelled by traveling‐wave deformation of millimeter‐thin pectoral fins.Reproduced with permission [82]. Copyright 2025, American Association for the Advancement of Science. (F) Multi‐directional water walking via drag‐based backward kicking in a tri‐legged soft robot under photothermal actuation. Reproduced with permission [77]. Copyright 2022, Elsevier Ltd. (G) Floating state of a water‐surface‐jumping robot combining a superhydrophobic body with ultrafast actuation to escape the capillary barrier. Reproduced with permission [89]. Copyright 2025, Springer Nature. (H) A piezoelectrically actuated multimodal flapping‐wing micro‐robot whose adjustable wing attack angle enables floating, forward swimming, yawing, and pitching. Reproduced with permission [93]. Copyright 2025, American Association for the Advancement of Science. (I) Flatfishbot maneuvering underwater by controlling localized buoyancy with thermoelectric phase‐change actuators. Reproduced with permission [96]. Copyright 2022, Wiley‐VCH GmbH. (J) Untethered swimming driven by a jelly‐like dielectric‐elastomer (DE) artificial muscle, with reversible direction switching through voltage‐waveform asymmetry. Reproduced with permission [98]. Copyright 2024, Elsevier Inc. (K) Snailfish‐inspired self‐powered soft robot with distributed printed‐circuit units encapsulated in a silicone matrix, demonstrating pressure tolerance through avoidance of stiffness discontinuities. Reproduced with permission [101]. Copyright 2021, Springer Nature. (L) Schematic of a deep‐sea field test by a plasticized electrohydraulic robot at 4,071 m in the South China Sea. Reproduced with permission [106]. Copyright 2025, American Association for the Advancement of Science.

A fundamental requirement for all aquatic soft robots is reliable waterproof sealing. Water incursion can damage onboard electronics, reduce buoyancy, and, in fluid‐driven systems, disable actuation by depleting the working medium [15, 70]. Established sealing strategies include rigid shell encapsulation [71, 72], conformal polymer coatings [73], and flexible skin coverings [74], with the choice depending on the required compliance and operating depth. Beyond ensuring survivability, aquatic soft robots offer functional advantages for low‐disturbance observation. Their compliant bodies and distributed propulsion generate substantially weaker hydrodynamic disturbances than rigid jet‐propelled vehicles, enabling closer interaction during in situ monitoring [14, 75]. Together, reliable waterproofing and low hydrodynamic disturbance have become fundamental design considerations for aquatic soft robots.

At the air‐water interface, locomotion is governed primarily by capillary support rather than buoyancy, and only robots whose weight remains below the relevant capillary limit can stably operate on the free surface (Figure 2D) [76]. Successful operation therefore depends on maintaining a stable non‐wetting state while generating sufficient propulsion without disrupting the supporting interface. Superhydrophobic surface treatments, typically achieved through nanostructured roughness combined with low‐surface‐energy coatings, are widely used to preserve capillary support [77, 78, 79, 80, 81]. On this basis, locomotion strategies can be grouped into three broad categories: direct mechanical interaction with the water surface, propulsion through modulation of interfacial forces, and transient escape from the interface. For direct mechanical propulsion, Hartmann et al. developed a centimeter‐scale robot with millimeter‐thin soft pectoral fins, in which electrically excited traveling waves generated thrust at the interface (Figure 2E) [82]. Yang et al. demonstrated drag‐based momentum generation through backward kicking (Figure 2F) [77], whereas Zhang et al. achieved propulsion through flexible undulation of a magnetically driven sheet [83]. Elastocapillary propulsors provide another route by using passive structural deployment upon water contact to enhance thrust and maneuverability [76], while Marangoni propulsion uses surface‐tension gradients induced by localized heating [84] or surfactant release [85] to drive motion without dedicated appendages. Ke et al. extended this interfacial approach with a Stenus‐inspired soft propulsor that used magnetic tail deformation to regulate surfactant delivery [86]. Surface‐tension forces and flow‐mediated momentum transfer produced propulsion, while magnetic steering and rapid posture changes enabled turning and braking. A 3.6 mm device reached a reported peak speed of approximately 201 BL/s. This example shows that useful surface locomotion requires control of starting, steering, and stopping as well as high speed, and that propulsion and steering can be supplied through different physical mechanisms. Beyond propulsion along the interface, reversibly switchable hydrophobicity also enables controlled transitions between surface and submerged operation [87, 88]. Escaping the interface altogether requires transient forces that exceed the capillary barrier. Wang et al. addressed this challenge with an insect‐scale jumper that combined a superhydrophobic body with an ultrafast SMA actuation module, achieving a take‐off velocity of 3.52 m/s and a jumping height of 63 cm (Figure 2G) [89]. Overall, water‐surface soft robots operate within a tightly constrained design space, in which successful locomotion depends on optimizing mass distribution, wetting state, and actuation timescale.

As robots transition below the water surface, capillary effects rapidly become negligible and hydrodynamic drag becomes the dominant constraint on locomotion. Now, the primary design objectives shift toward effective thrust generation, maneuverability, and depth control. Propulsion architectures for soft swimmers can be grouped according to how thrust is generated and distributed across the body. Designs dominated by body undulation generally favor higher thrust and swimming speed, whereas designs dominated by fin oscillation typically provide better stability and maneuvering accuracy at lower speed. Marchese et al. demonstrated a body‐undulation approach with a fluidically actuated robotic fish, whose agonist‐antagonist tail channels produced continuum body motion at 0.44 body lengths per second (BL/s) [90]. Li et al. developed a soft robot driven by pectoral fins, using dielectric elastomer membranes as distributed actuators [91]. By utilizing the surrounding water as an electrical ground, the design avoided bulky high‐voltage insulation while achieving swimming speeds of 0.69 BL/s. Wang et al. reported a self‐powered robotic fish equipped with a 17.2 g fin that generated a peak thrust of 493 mN and enabled a maximum swimming speed of 1.66 BL/s [92]. At smaller scales, oscillatory appendage propulsion can also be implemented through flapping‐wing configurations. Liu et al. developed RoboPteropod, a piezoelectrically actuated flapping‐wing micro‐robot whose actively adjustable wing attack angle enabled floating, forward swimming, yawing, and pitching at a power consumption of only 580 mW (Figure 2H) [93]. These propulsor architectures therefore reflect a trade‐off between thrust generation and maneuvering stability, and the optimal choice depends on whether the mission prioritizes rapid transit, station‐keeping, or exploration within confined spaces.

Achieving full three‐dimensional mobility further requires effective depth control. Active buoyancy regulation provides a direct strategy because it modifies the net vertical force without requiring continuous thrust generation. Katzschmann et al. used linear actuators to compress or inflate an air chamber while simultaneously shifting the buoyant center for pitch adjustment [94]. However, the integrated gear motors hindered miniaturization and generated undesirable acoustic noise. Phase‐change strategies provide a pump‐free alternative. Kang et al. employed three inflatable pouches filled with low‐boiling‐point fluid, whose selective heating enabled multimode buoyancy adjustment [95]. Lee et al. further improved cycling speed by adding thermoelectric devices for active condensation (Figure 2I) [96]. At microscale, Guo et al. showed that electrolysis‐generated gas bubbles could modulate buoyancy, although the limited volume change restricted this strategy to very small platforms [97]. Rather than modifying buoyancy directly, some systems achieve depth control through propulsion asymmetry or impulsive body motion. Li et al. developed a dielectric‐elastomer artificial muscle whose extremely soft and highly aqueous structure enabled reversible switching of propulsion direction through voltage waveform asymmetry without requiring an additional steering mechanism (Figure 2J) [98]. An untethered version reached 0.91 cm/s, operated for 15.7 min on a 500 mAh battery, and retained repairability after both mechanical and electrical damage. Complementary to dielectric‐elastomer‐based approaches, Frame et al. systematically characterized pneumatic jellyfish robots and identified optimal combinations of material Shore hardness, stroke amplitude, and frequency for maximizing thrust, with ocean‐tested prototypes demonstrating untethered steering and passage through orifices narrower than the bell diameter [99]. At the opposite end of the force‐time spectrum, He et al. introduced a combustion‐driven Copebot capable of generating a peak impulsive acceleration of 850 BL/s^2^ and executing full 360° rotation during a single jump [100]. Thus far, these systems span locomotion strategies ranging from quasi‐steady buoyancy regulation to rapid impulsive repositioning, with the optimal approach determined by the trade‐off among precision, response speed, structural complexity, and energy cost.

The deep sea, below approximately 1000 m, imposes constraints that are qualitatively different from those encountered in shallower aquatic environments. In this regime, resilience to extreme hydrostatic pressure and low temperature takes precedence over locomotor agility. The resulting design framework must therefore jointly address pressure‐tolerant structures, cold‐resistant actuation, and electronics integration strategies that avoid catastrophic local stress concentration [5]. For electronics protection, a key principle is to avoid embedding large rigid inclusions within a compliant matrix, because stiffness discontinuities can amplify interfacial shear stress under pressure. Li et al. implemented this principle by partitioning the onboard printed circuit board (PCB) of a soft robot into smaller, wire‐connected units encapsulated in a silicone matrix (Figure 2K) [101]. Finite‐element analysis further confirmed that increasing the spacing between units lowered the average shear stress. For actuation, the dominant challenge is that low temperatures and high pressures drive polymeric materials toward glassy behavior, degrading the strain output of soft actuators [102, 103, 104, 105]. Li et al. addressed this issue through industrial plasticization. In their electrohydraulic soft robot, poly‐α‐olefin simultaneously softened the polyethylene shell and served as the working fluid, enabling untethered swimming and maneuvering at depths of up to 4071 m in the South China Sea (Figure 2L) [106]. These deep‐sea examples illustrate that pressure‐tolerant soft robots require the co‐design of distributed structures, plasticized materials, and compliant electronics integration, rather than relying solely on rigid pressure vessels.

Aquatic navigation requires information about depth, attitude, heading, and motion relative to the surrounding flow. The sensing and communication architecture must be considered separately from the source of propulsion. In Katzschmann et al.’s soft robotic fish, acoustic communication transmitted operator commands, while hydraulic actuation generated swimming motion [94]. This distinction is important because remote command reception does not itself demonstrate autonomous navigation or acoustic propulsion. For feedback‐controlled operation, the reported sensing arrangement and control bandwidth should be related to the disturbances encountered, including whether depth and heading can be maintained without repeated human correction.

### 2.3 Aerial Soft Robots

The aerial environment imposes exceptionally stringent constraints on soft robot design. Aerial systems must obtain adequate aerodynamic or buoyant support, or regulate descent, within a restricted mass and energy budget. At low altitude, unsteady wind fields can strongly perturb lightweight soft flyers, demanding effective gust rejection and flight stabilization [107, 108]. Precipitation and humidity can further alter effective mass and aerodynamic performance [109]. At higher altitudes, air density drops sharply, approximately 33% at 4000 m relative to sea level, reducing aerodynamic lift [110]. Meanwhile, lower temperatures can further degrade actuator performance [111, 112, 113]. We group aerial examples by how weight support and motion are achieved: powered aerodynamic flight, buoyancy‐assisted flight, and passive or weakly actuated dispersal. The first group includes flapping flight and related flight technologies discussed for their power and integration relevance. Altitude modifies the constraints on each regime but is not itself the basis of this classification.

Aerial success criteria depend on the flight regime. For powered aerodynamic flight, upward acceleration requires the vertical component of the net aerodynamic force to exceed weight, whereas steady hovering requires average vertical force balance. Evaluation should distinguish take‐off from sustained controlled flight and report total flight mass, payload fraction, power consumption, flight duration, and attitude or position error. Buoyancy‐assisted systems should additionally report the contribution of buoyancy to weight support. Passive dispersal is instead assessed through descent speed, airborne residence time, trajectory repeatability, and landing error under specified release and airflow conditions.

Flapping‐wing propulsion remains the primary strategy for insect‐ and bird‐scale aerial soft robots because a single compliant wing system can simultaneously generate lift, thrust, and control torque under low‐Reynolds‐number conditions (Figure 3A) [114, 115, 116]. Although most current demonstrations remain tethered, these studies provide important design guidance for future untethered development. Within this framework, actuator selection becomes the central design variable and is governed by a trade‐off among strain amplitude, operating bandwidth, force output, and system mass. Dielectric elastomer actuators (DEAs) offer large strain and intrinsic compliance. Chen et al. demonstrated a DEA‐powered insect robot using Elastosil P7670, an elastomer with low viscoelasticity and high dielectric strength, achieving a power density of 1.2 kW kg^−1^ and a lift‐to‐weight ratio of 2.2 [117]. Piezoelectric actuators, by contrast, provide high bandwidth and power density but require mechanical amplification because of their limited intrinsic strain. James et al. developed a honeybee‐sized robot whose bimorph cantilever transmission amplified piezoelectric strain to a 90° wing stroke, requiring only 250 mW wirelessly delivered by a laser‐illuminated photovoltaic cell [118]. Electromagnetic actuators provide a third option, offering high force at the cost of increased mass. For example, Zou et al. used a four‐bar linkage to convert small‐amplitude electromagnetic oscillations into wing strokes exceeding 70° [119]. A different strategy is to eliminate the transmission altogether, thereby reducing mechanical complexity and transmission loss. Helps et al. developed a liquid‐amplified zipping actuator that applied electrostatic forces directly to the wing root through a retained liquid dielectric, achieving more than one million actuation cycles without performance degradation [120]. Across these systems, a recurring design trade‐off is evident: DEAs provide the largest strains but require high driving voltages, piezoelectrics offer the highest bandwidth but depend on mechanical amplification, and electromagnetic actuators deliver high force but at greater mass. At present, no single actuator technology satisfies all the requirements for efficient, untethered, insect‐scale flapping flight [16, 21, 121, 122, 123, 124].

**FIGURE 3:**
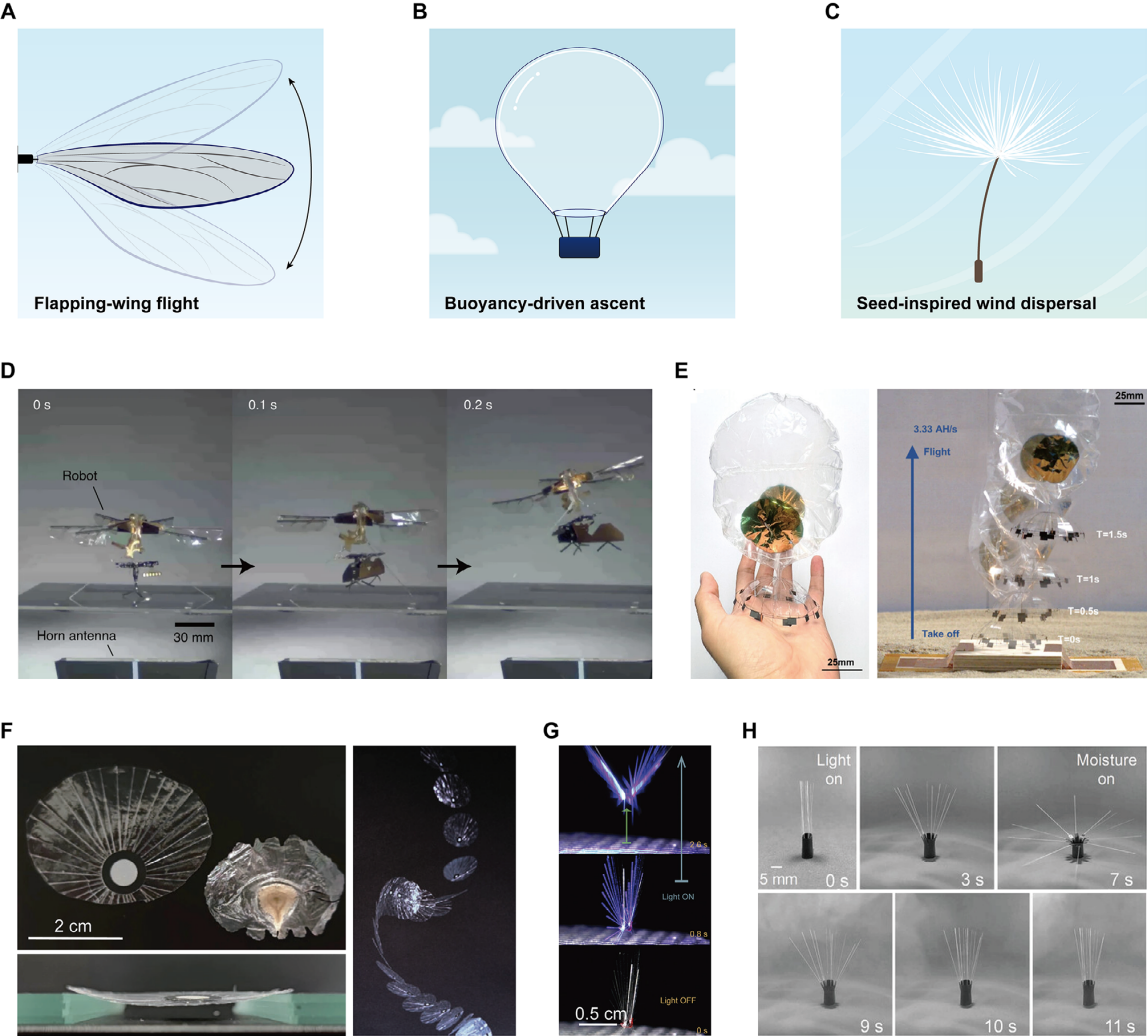
Aerial soft robotic systems and enabling technologies under mass and power constraints. The examples are organized by powered aerodynamic flight, buoyancy‐assisted flight, and passive or weakly actuated dispersal. (A) Schematic of flapping‐wing flight. (B) Schematic of buoyancy‐driven ascent flight. (C) Schematic of seed‐inspired wind dispersal. (D) Wireless radiofrequency‐powered piezoelectric aerial vehicle with sub‐gram receivers achieving high power density. Reproduced with permission [125]. Copyright 2021, Springer Nature. (E) Take‐off and flight of a helium‐balloon micro‐robot powered by electrostatic flapping. Reproduced with permission [128]. Copyright 2022, Wiley‐VCH GmbH. (F) Rapid rotational descent of an ultrathin microflier with heterogeneous venation patterns and thickness gradients in a turbulent flow field. Reproduced with permission [132]. Copyright 2026, Oxford University Press on behalf of China Science Publishing & Media Ltd. (G) Light‐responsive LCE assembly enabling on‐demand landing of an artificial seed without onboard electronics. Reproduced with permission [134]. Copyright 2022, Wiley‐VCH GmbH. (H) Dandelion‐inspired artificial seed with piezoresistive self‐sensing for real‐time deformation monitoring under infrared and humidity stimuli. Reproduced with permission [135]. Copyright 2024, Wiley‐VCH GmbH.

A closely related challenge is sustaining flight without a tether, which requires reliable onboard energy delivery under extreme mass constraints. Jafferis et al. achieved the first sustained untethered flight of an insect‐scale vehicle by directly integrating onboard photovoltaics with piezoelectric actuators, although the system required illumination at approximately three times solar intensity [6]. Ozaki et al. showed that this energy bottleneck could also be addressed through far‐field radiofrequency power transfer, demonstrating untethered take‐off of a 1.8 g piezoelectric flapping‐wing vehicle using a sub‐gram radiofrequency power receiver with a power density of 4900 W kg^−^^1^ (Figure 3D) [125]. As a related power‐integration example, Shen et al. demonstrated sustained sunlight‐powered flight under natural illumination with CoulombFly, a 4.21 g electrostatic rotorcraft that achieved a lift‐to‐power efficiency of 30.7 g W^−^^1^ [126]. Beyond exploring alternative power‐supply pathways, untethered operation can also be supported by reducing the mechanical overhead of flight. Phan et al. showed that passive wing deployment and rapid retraction could eliminate additional deployment actuators in a flapping micro‐robot, illustrating how morphological simplification can reduce both structural mass and power consumption while maintaining self‐contained operation [127]. These studies suggest that progress toward untethered aerial soft robots depends not only on improving onboard energy delivery, but also on reducing the structural and actuation demands imposed on limited power resources.

Buoyancy‐assisted systems provide an energy‐efficient alternative means of weight support and can reduce the power needed to remain aloft, although their size and payload capacity still depend on ambient density and gas volume (Figure 3B). Helium‐filled platforms remain aloft with minimal energy expenditure, requiring onboard actuation mainly for propulsion, steering, and altitude adjustment. Yun et al. developed a helium micro‐robot with electrostatic flapping that achieved untethered ascent to 2 m (Figure 3E) [128]. Jordi et al. demonstrated dielectric‐elastomer actuated undulatory propulsion on a helium airship [129]. Chen et al. introduced a robotic gliding blimp equipped with a bird‐inspired continuum arm and a moving‐mass actuation mechanism for glide control [130]. Yamada et al. proposed a blade‐free blimp steered by piezoelectric microblowers [131]. Compared with flapping‐wing robots, buoyancy‐assisted systems sacrifice speed and agility but substantially reduce the energetic cost of sustained aerial operation, making them attractive for long‐duration monitoring and atmospheric sensing.

In contrast to powered aerial strategies, a growing class of soft aerial systems reduces energy demand by exploiting passive or hybrid aerodynamic mechanisms for descent, dispersal, and limited aerial control. The central design objective in this regime is to maximize airborne residence time or achieve controlled descent without continuous onboard actuation. One approach is to tailor ultralight wing or membrane structures so that drag, autorotation, or passive aeroelastic deformation stabilizes flight (Figure 3C). For example, Wu et al. showed that heterogeneous venation patterns and thickness gradients in ultrathin wing structures can produce a favorable combination of flexural rigidity and aerodynamic performance (Figure 3F) [132]. Kim et al. translated related principles into three‐dimensional electronic microfliers with battery‐free wireless sensing, enabling controlled rotational descent for distributed environmental monitoring [133]. Passive flight can also be extended toward limited controllability through environmentally responsive structures. Yang et al. developed a light‐responsive LCE assembly that enabled on‐demand landing control without onboard electronics (Figure 3G) [134]. Yan et al. further incorporated piezoresistive self‐sensing for real‐time deformation monitoring during flight (Figure 3H) [135]. These passive and hybrid approaches broaden the aerial design space by trading maneuvering agility for lower energy consumption, extended flight endurance, and wider environmental coverage.

Aerial control must operate within the mass, power, and response‐time limits of the complete flight system. Force generation alone does not demonstrate stable navigation: sustained controlled flight also requires attitude regulation and, where claimed, position or trajectory feedback. Reports should identify whether measurements and commands originate onboard, from external tracking, or from an operator. Passive aeroelastic stabilization and stimulus‐triggered landing can reduce control demands, but do not establish autonomous route selection. The mass and power required by sensors, receivers, computation, and control electronics should therefore be included when assessing the demonstrated flight capability.

### 2.4 In Vivo Soft Robots

The in vivo environment imposes coupled geometric, mechanical, chemical, and immunological constraints on untethered soft robots. Anatomical pathways such as blood vessels, gastrointestinal tracts, bronchial passages, and interstitial spaces are typically narrow and tortuous, while the surrounding tissues are mechanically fragile and exhibit substantial variations in stiffness across organs and length scales [17, 18, 136, 137]. Robots operating in these environments must also tolerate continuous physiological motion, chemically heterogeneous local conditions, and host immune responses that can trigger inflammation, fibrotic encapsulation, or rejection [138, 139, 140]. These constraints define core design requirements: extreme miniaturization combined with high compliance, biocompatibility and immunological safety, wireless and remotely controllable actuation, and precise cargo management with on‐demand release. In vivo constraints act together rather than varying along a single environmental coordinate. We therefore organize this section by the functions they restrict: wireless actuation through tissue, navigation under confinement and flow, and cargo handling or release at the target. This sequence links the actuation route to the subsequent requirements for completing a biomedical task.

Successful operation for an in vivo task requires navigation to the intended location and completion of the specified action under relevant anatomical and physiological conditions. Evaluation should report localization or targeting error, completion rate and time, payload delivery or release efficiency, and operating duration, together with lumen dimensions, flow conditions, and evidence of tissue compatibility. Phantom, ex vivo, and in vivo demonstrations should be identified separately. A navigation or delivery result establishes performance under its tested conditions; it does not alone establish broader biological safety or therapeutic effectiveness.

Among these requirements, wireless actuation is fundamental because robots must be controlled through opaque and confined biological tissue without tethered connections or bulky onboard power systems. Magnetic actuation remains the most widely used strategy, because external magnetic fields penetrate tissue relatively deeply and noninvasively and enable precise remote steering without onboard power [141]. Hu et al. developed a magneto‐elastic soft millirobot capable of multimodal locomotion under magnetic fields [142], while Xu et al. showed that programmable 3D magnetic soft machines can simultaneously support controlled locomotion and structural reconfiguration in miniature biomedical settings (Figure 4A,B) [143]. Acoustic actuation provides a complementary wireless modality for rapid structural reconfiguration and propulsion within confined biofluidic environments. Representative examples include acoustically activated transformable micromachines with millisecond‐scale folding and rotational motion [144], as well as bioresorbable acoustic hydrogel micro‐robots capable of stable propulsion in biological fluids with real‐time ultrasound tracking [145]. Biohybrid systems offer another untethered route by embedding propulsion within living cells or microorganisms. Alapan et al. constructed erythrocyte‐based bacterial micro‐swimmers with onboard bacterial propulsion and magnetic steering [146]. Xu et al. developed a sperm‐hybrid micro‐motor integrating cell‐based propulsion with magnetic guidance and contact‐triggered cargo release [147]. Zhang et al. demonstrated algae‐based micro‐robots with sustained autonomous motility and intrinsic fluorescence tracking under physiological lung conditions [148]. Taken together, in vivo wireless actuation follows two main routes: externally applied fields for precise remote control, and biohybrid systems that embed propulsion within living carriers.

**FIGURE 4:**
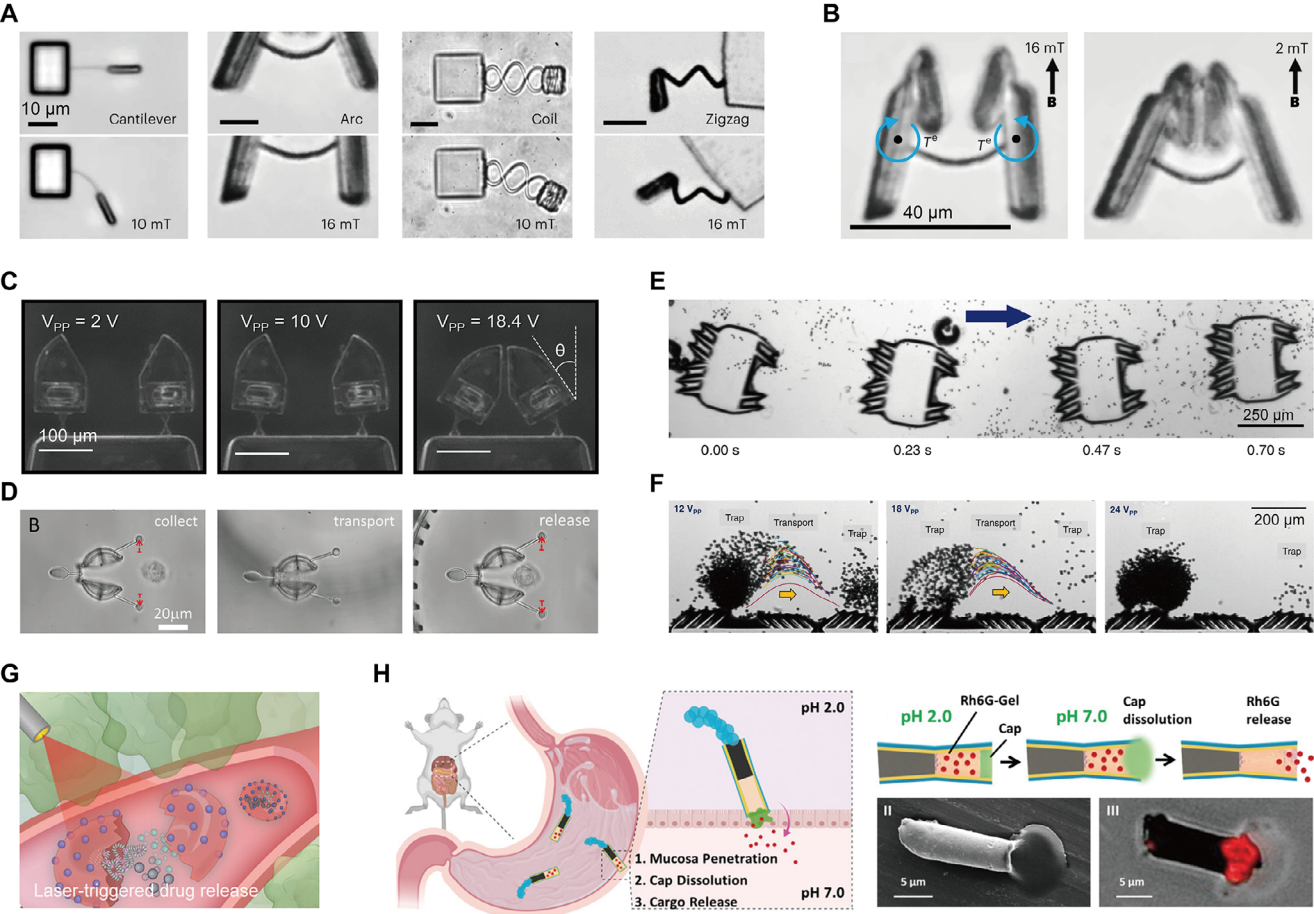
Soft robotic systems and enabling studies for in vivo applications, organized by wireless actuation, navigation, and cargo‐delivery function. Tissue‐penetrating fields and biohybrid carriers each provide tether‐free control, while cargo delivery proceeds through either active transport of an external load or programmed release of a body‐integrated payload. (A) Programmable 3D magnetic soft micro‐robot with super‐compliant picoforce springs serving simultaneously as onboard sensors and actuators under magnetic loading [143]. (B) Opening and closing images of the microgripper. Reproduced with permission [143]. Copyright 2024, Springer Nature. (C) Acoustically powered micro‐clampbot whose bubble‐actuated claws close in response to applied voltage for single‐particle pick‐and‐place transport through confined channels. Reproduced with permission [155]. Copyright 2025, American Association for the Advancement of Science. (D) Microscopic sequence showing optical trapping, transport, and gentle release of single cells by an elastic microtool. Reproduced with permission [156]. Copyright 2024, Wiley‐VCH GmbH. (E) Time‐lapse superposition of acoustic‐streaming‐mediated translation by starfish‐inspired ciliary bands [157]. (F) Acoustic‐power‐dependent transport and trapping in source‐ and sink‐like streaming fields generated by the same ciliary‐band system. Reproduced with permission [157]. Copyright 2021, Springer Nature. (G) Schematic of laser‐triggered drug release from an RBC‐derived micro‐robot accumulated under magnetic guidance. Reproduced with permission [159]. Copyright 2023, Henan University and John Wiley & Sons Australia, Ltd. (H) Schematic of a multicompartment tubular micro‐motor with a pH‐responsive cap, enabling stable gastric propulsion followed by autonomous localized release after mucosal penetration. Reproduced with permission [161], et al., Copyright 2020, WILEY‐VCH Verlag GmbH & Co. KGaA, Weinheim.

Within these wireless control constraints, successful navigation through tortuous and mechanically fragile anatomical pathways requires a balance between tissue‐matched compliance and sufficient structural integrity for task execution [149, 150]. At the millimeter scale, representative systems traverse lumen‐like spaces through large deformation, crawling, or geometry‐adaptive locomotion. Lu et al. developed a multi‐legged PDMS/iron crawler capable of transporting a capsule twice its weight in a human stomach model [151]. Ren et al. showed that a magnetically actuated soft sheet could use confinement to obtain traction as well as adapt its shape [152]. Traveling body waves produced crawling through alternating edge anchoring at the channel walls. When viscous and frequency‐dependent effects reduced the undulation amplitude and weakened anchoring, swimming could become dominant. In cylindrical passages, a helical configuration instead used wall contact for translation and resistance to flow. Switching among these modes enabled passage through a fluid‐filled Eustachian‐tube phantom with changing cross sections. The result links locomotion selection to geometry, fluid properties, and available contact, rather than to robot size alone. A limitation of many magnetically actuated systems is that their magnetization profiles are fixed during fabrication, which restricts the range of accessible deformation modes. Bao et al. addressed this constraint through real‐time in situ magnetization reprogramming, enabling a single robot to adapt its deformation pattern and perform multiple tasks under a single external field [153].

Vascular navigation additionally requires maintaining position or advancing against flow while avoiding excessive loading of the vessel wall. Liu et al.’s magnetic soft microfiberbots combined wall anchoring with helical propulsion and reversible elongation or aggregation [154]. Elongation allowed passage through constrictions, whereas aggregation supported embolization at the target. Navigation and embolization were demonstrated in neurovascular phantoms and a rabbit femoral artery model, with fluoroscopic guidance for the in vivo procedures. Together, these studies indicate that geometric passability is insufficient to establish navigational capability: evaluation should also specify opposing‐flow tolerance, turning and branch‐selection performance, localization accuracy, and the consequences of wall contact. Test fluids and wall mechanics should represent the intended setting, and unintended obstruction should be distinguished from deliberate target occlusion.

Beyond wireless actuation and navigation, in vivo soft robots must also implement reliable cargo delivery. In current designs, this is generally achieved through two distinct strategies. One strategy is the active transport of an external cargo, which is typically implemented through either direct capture or flow‐mediated manipulation. Miao et al. developed an acoustically powered micro‐clampbot whose bubble‐actuated claws clamp individual particles or cells while acoustically driven flagella provide locomotion through confined channels, enabling precise pick‐and‐place transport at the microscale (Figure 4C) [155]. A conceptually related but mechanically softer approach was demonstrated by Iványi et al., who developed optically actuated elastic microtools that could enclose, transport, and release single cells without significant squeezing or permanent biochemical attachment (Figure 4D) [156]. A different approach is flow‐mediated transport. Dillinger et al. showed that ultrasound‐activated ciliary bands could generate source‐ and sink‐like streaming fields for microparticle transport and trapping without direct mechanical gripping (Figure 4E,F) [157]. Ghadami and Shum extended this non‐contact route to filamentous cargo by designing a magnetic helical micro‐robot whose alternating pulling and pushing sections hydrodynamically captured and transported stiff filaments without requiring direct contact, thereby reducing adhesion‐related release difficulties [158]. The second strategy integrates the therapeutic payload into the robot body and releases it only after localization. Zhu et al. developed a red blood cell (RBC)‐derived micro‐robot that preserved RBC‐like circulation, accumulated under magnetic guidance, and underwent laser‐triggered disruption to release its cargo locally (Figure 4G) [159]. Ha et al. further demonstrated phototactic biohybrid microalgae coated with drug‐loaded peptide nanotubes that maintained active swimming and phototaxis but disassembled under acidic tumor‐like conditions to release the payload [160]. A related pH‐responsive strategy was reported by Esteban–Fernández de Ávila et al., who developed a multicompartment tubular micro‐motor whose cargo segment was sealed by a pH‐sensitive cap, allowing stable gastric propulsion followed by autonomous localized release after penetration into the gastric mucosa (Figure 4H) [161]. This distinction separates cargo delivery into external‐cargo handling and body‐integrated payload delivery: the former relies on direct capture or flow‐mediated transport of a discrete cargo, whereas the latter stores the therapeutic payload within the robot and releases it only after localization.

At biomedical scales, localization is often provided externally. Han et al., for example, combined ultrasound‐visible hydrogel microrobots with acoustic propulsion and magnetic navigation [145]. An imaging‐guided system should report how localization informs actuation, including whether updates are generated automatically or supplied by an operator. Imaging resolution, update rate, localization error, and accessible depth constrain the achievable feedback performance. Stimulus‐responsive motility or payload release can support a task, but evidence of autonomous navigation additionally requires a demonstrated link between observation, selection of an action, and correction of the resulting motion.

## 3 Actuation Strategies and Quantitative Performance Across Environments

The actuation routes discussed here differ in how energy reaches the robot and how it is converted into force or deformation. These two stages should be distinguished: light may directly deform a responsive material or supply electricity through photovoltaics, and an acoustic link may transmit commands without producing thrust. Habitat suitability depends on the complete pathway, including transmission through the surrounding medium, mechanical transduction, and the infrastructure needed to sustain operation. To map these relationships, we classified 100 representative studies according to their actuation modality and operating environment (Figure 5). Only systems with demonstrated untethered operation or wireless power/actuation were included in the classification. To complement the habitat‐specific case studies in Section 2, we compiled a structured quantitative comparison of representative untethered soft robotic systems across the four operating habitats (Table 1). The comparison includes system size and mass, actuation modality, power source and energy capacity, operating duration, absolute and size‐normalized locomotion speed, payload capacity, environmental/test conditions, and reported limitations or failure modes. Parameters unavailable in the original studies are explicitly marked as “NR”. This multidimensional comparison complements the actuation‐distribution analysis in Figure 5 and the locomotion‐performance map in Figure 6, allowing the performance of individual systems to be interpreted together with their scale, energy supply, endurance, task requirements, and environmental constraints.

**FIGURE 5:**
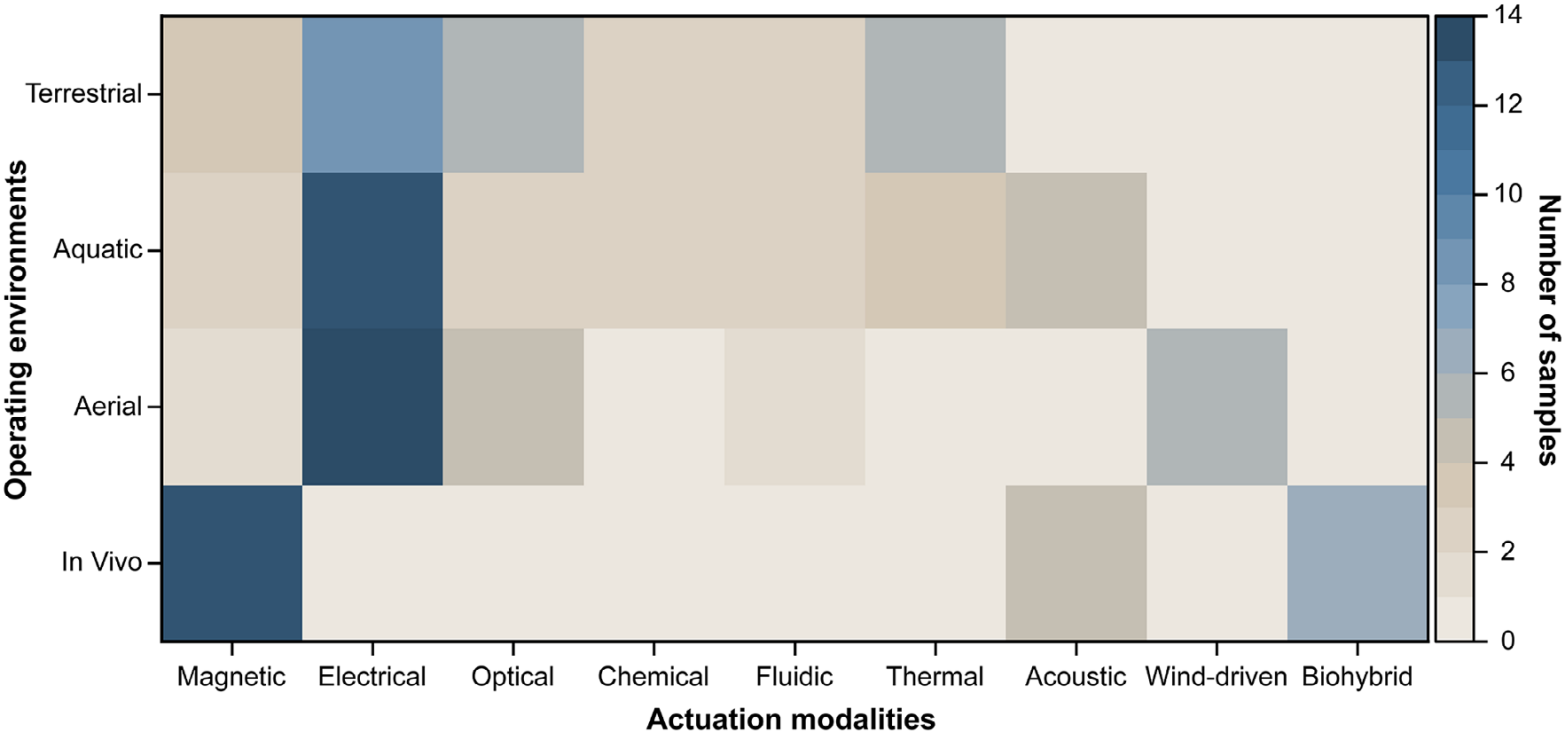
Distribution of representative untethered soft robotic systems across actuation mechanisms and operating environments. A heatmap classifying representative studies by nine actuation modalities (magnetic, electrical, optical, chemical, fluidic, thermal, acoustic, wind‐driven, and biohybrid) and four operating environments (terrestrial, aquatic, aerial, and in vivo). Color intensity encodes the number of samples per cell, with deeper blue indicating higher prevalence. Three patterns emerge from the distribution. First, the operating environment acts as a selective filter on actuation choice: terrestrial settings accommodate the broadest modality range, whereas aerial and in vivo settings concentrate around a narrower set of mechanisms. Second, certain modalities are nearly habitat‐specific, most strikingly wind‐driven actuation, which is essentially unique to aerial soft robots, and biohybrid actuation, which is largely confined to in vivo systems. Third, the dominant modality shifts qualitatively between habitats: electrical actuation prevails across terrestrial, aquatic, and aerial systems but is largely absent in vivo, where magnetic actuation predominates because magnetic fields can penetrate biological tissue without requiring onboard power.

**TABLE 1:** Quantitative comparison of representative untethered soft robots across operating habitats.

A. Terrestrial environments (Section 2.1)			
System and reference	Architecture and energy	Quantitative performance	Test conditions/limitations
Piezoelectric crawler [39]	Size: 2.4 cm × 2.2 cm Mass: 240 mg Actuation: Piezoelectric body; electrostatic feet Power source: Onboard battery Capacity/input: 3.7 V, 40 mAh	Absolute speed: 7.56 mm/s Normalized speed: 0.315 BL/s Operating duration: 36.9 s Payload/ratio: 1660 mg	Test conditions: Untethered loaded configuration; S‐shaped path Limitations: 65 mg and faster tethered results excluded
Pneumatic quadruped [40]	Size: Length 650 mm Mass: NR Actuation: Pneumatic limbs Power source: Onboard compressors and electrical supply Capacity/input: 3200 mAh	Absolute speed: >18.0 m/h Normalized speed: 0.0077 BL/s Operating duration: 2 h Payload/ratio: NR	Test conditions: Flat‐ground walking Limitations: Separate 2.2 kg moving‐load and 8 kg static‐load tests are not assigned to the untethered configuration
Graded combustion jumper [43]	Size: ∼10 cm height Mass: 964.6 g Actuation: Butane‐oxygen combustion Power source: Onboard fuel and ignition/control battery Capacity/input: 7.4 V, 180 mAh control battery	Absolute speed: 2 m/s Normalized speed: 18 BL/s Operating duration: 21 successful untethered jumps Payload/ratio: NR	Test conditions: Untethered jumping on solid substrate Limitations: Cumulative jump count is not single‐charge endurance; 89 additional tethered jumps excluded
LCE–CNT crawler and jumper [46]	Size: 40 mm × 7 mm × 2 mm Mass: NR Actuation: Photothermal deformation and snap‐through Power source: External light Capacity/input: Crawling irradiance 1.57 W/cm^2^	Absolute speed: 0.7 mm/s crawling Normalized speed: 0.0083 BL/s Operating duration: NR Payload/ratio: NR	Test conditions: Illuminated substrate and confined‐channel tests Limitations: Crawling and jumping use different illumination programs; no shared maximum operating point
SMA soft locomotors [54]	Size: 60 mm Mass: 3 g refers to one limb Actuation: SMA wires in thermally conductive elastomer Power source: Onboard power and control Capacity/input: 7.4 V, 180 mAh control battery	Absolute speed: 32 mm/s Normalized speed: 0.56 BL/s Operating duration: NR Payload/ratio: 30 g	Test conditions: Level, inclined, and rocky surfaces Limitations: Higher peak speeds risk overheating; do not import caterpillar endurance or payload
HSA quadrupeds [205]	Size: 280 × 280 × 200 mm Mass: About 2 kg; varies with leg design Actuation: Servo‐driven TPU handed‐shearing‐auxetic legs Power source: Onboard batteries and microcontroller Capacity/input: 7.4 V, 5000 mAh; 37 Wh nominal	Absolute speed: 183 mm/s Normalized speed: 0.65 BL/s Operating duration: 5‐min trials Payload/ratio: NR	Test conditions: Flat surface; fixed‐axis circular guidance Limitations: Speed measured under mechanical constraint
LCN transporter [206]	Size: 20 mm Mass: 20 mg Actuation: Photoresponsive LCN legs, arms and gripper Power source: External blue light Capacity/input: 455 nm; 150–200 mW/cm^2^	Absolute speed: 0.5 mm/s Normalized speed: 0.025 BL/s Operating duration: NR Payload/ratio: 5 mg	Test conditions: Paper substrate; typical stride about 4 mm Limitations: No locomotion on tested glass or sandpaper; coordinated illumination required
Phase‐transition robot [207]	Size: 30 mm × 7 mm × 0.25 mm Mass: 0.36 g for jumper Actuation: Light‐heated phase‐change chamber; elastic release; photothermal feet Power source: External light; onboard phase‐change liquid Capacity/input: Jumping: 808 nm, 1.25 W/cm^2^; crawling: 1.41 W/cm^2^	Absolute speed: 0.11 mm/s; Normalized speed: 0.0037 BL/s Operating duration: 290 s crawling sequence Payload/ratio: NR	Test conditions: Paper crawling surface; separate jumping test Limitations: Takeoff speed/height require consistency clarification; fixed launcher throwing 1.8 g is not a mobile payload
LCE tubular‐leg robot [210]	Size: 63 mm Mass: NR Actuation: Joule‐heated LCE tubular actuators Power source: Onboard LiPo and Arduino Mini Capacity/input: 3.7 V, 1300 mAh	Absolute speed: 0.06 mm/s Normalized speed: 0.00095 BL/s Operating duration: 30 min Payload/ratio: 10 g	Test conditions: Untethered walking and object transport under laboratory conditions Limitations: slow cooling limits actuation frequency and locomotion speed
SEMR UR1‐terrestrial [211]	Size: 20 mm Mass: 2.2 g Actuation: Soft electromagnetic actuation Power source: Onboard LiPo/PCB; external static magnetic field Capacity/input: 40 mAh	Absolute speed: Running 42 mm/s Normalized speed: 2.1 BL/s Operating duration: 400 s Payload/ratio: NR	Test conditions: 3D‐printed track; deionized water Limitations: Two media are separate tests; do not use tethered 70 BL/s or 4.8 BL/s
Self‐oscillating two‐limbed robot [214]	Size: 90 mm Mass: 76.7 g Actuation: Pneumatic TPU pouch limbs Power source: Two onboard 3 V pumps Capacity/input: 3.7 V, 380 mAh LiPo	Absolute speed: 181 mm/s Normalized speed: 1.93 ± 0.07 BL/s Operating duration: NR Payload/ratio: NR	Test conditions: Flat ground and gravel; separate amphibious demonstration Limitations: 30 BL/s belongs to tethered four‐limbed robot; spontaneous obstacle turns are not accurate path tracking
PCP robotic dog [215]	Size: 250 × 170 × 300 mm Mass: 1.57 kg Actuation: Servo/tendon‐controlled precharged pneumatic soft legs Power source: Onboard Li‐ion; no onboard compressor Capacity/input: 7.4 V, 6000 mAh; 44.4 Wh nominal	Absolute speed: 230 mm/s Normalized speed: 0.97 BL/s Operating duration: NR Payload/ratio: Trotting 1.5 kg; static 5.5 kg	Test conditions: Maximum‐speed test: 100 kPa, 360 ms gait; separate 13° slope and 30 mm step‐down Limitations: High speed and 1.8 kg load cause instability. Static support is not a trotting payload; source static ratio differs
Six‐legged pneumatic walker [216]	Size: 37 cm Mass: 1,030.9 g including supply Actuation: Pneumatic legs and fluidic logic Power source: Onboard CO_2_ cartridge and regulator Capacity/input: 16 g CO_2_	Absolute speed: 41 mm/s Normalized speed: 0.11 BL/s Operating duration: 46.56 ± 12.1 s (SD) Payload/ratio: NR	Test conditions: Untethered cartridge‐powered configuration Limitations: Tethered 0.17 BL/s excluded; supply increases mass; sensor timing is not whole‐robot stopping time

B. Aquatic environments (Section 2.2)			
System and reference	Architecture and energy	Quantitative performance	Test conditions/limitations
Flat surface swimmer [82]	Size: ∼4.5 cm Mass: NR Actuation: Electrohydraulic fins Power source: Onboard power available Capacity/input: <500 V and 35 mW reported	Absolute speed: 51 mm/s Normalized speed: 1.13 BL/s Operating duration: NR Payload/ratio: Pushes objects >16× body mass	Test conditions: Water‐surface tests Limitations: Numerical maxima need association with the untethered configuration; pushed load is not carried load
Water‐surface jumper [89]	Size: ∼35 mm Mass: 266 mg jumper + 144 mg pad = 410 mg Actuation: Photothermal hydrogel impulse Power source: External optical heating Capacity/input: NR	Absolute speed: 3,520 mm/s takeoff inferred from jump dynamics Normalized speed: 100.6 BL/s takeoff Operating duration: NR Payload/ratio: NR	Test conditions: Water surface with superhydrophobic support pad Limitations: Takeoff speed is not swimming speed
Soft electronic fish [91]	Size: 93 mm Mass: 90.3 g Actuation: Dielectric elastomer fins Power source: Onboard battery and voltage converter Capacity/input: 3.7 V, 450 mAh	Absolute speed: 64 mm/s Normalized speed: 0.69 BL/s Operating duration: 3 h 15 min at 11 mm/s Payload/ratio: NR	Test conditions: Maximum speed at 9.5 kV, 5 Hz; separate endurance condition Limitations: 135 mm/s and 1.45 BL/s are tethered results; maximum speed and endurance not simultaneous
RoboPteropod [93]	Size: 75 mm × 40 mm × 45 mm Mass: 34 g Actuation: Piezoelectric flapping wings; adjustable attack angle Power source: Onboard battery and controller Capacity/input: 580 mW total power	Absolute speed: 48 mm/s Normalized speed: 1.2 BL/s Operating duration: NR Payload/ratio: NR	Test conditions: Laboratory multimodal swimming Limitations: Reported forward normalization implies 40 mm rather than 75 mm
SoFi [94]	Size: 470 × 230 × 180 mm Mass: 1.6 kg Actuation: Hydraulic tail and active buoyancy Power source: Onboard battery; acoustic commands Capacity/input: 35 Wh	Absolute speed: 235 ± 4 mm/s Normalized speed: 0.5 BL/s Operating duration: 40 min per dive; six dives reported Payload/ratio: Integrated camera	Test conditions: Coral reef; tested depths 0–18 m Limitations: Ballast adjustments for depth; compression limited upward diving at greater depth
Flatfishbot [96]	Size: 20 cm Mass: NR Actuation: Thermoelectric liquid–vapor buoyancy pouches Power source: Onboard battery and controller Capacity/input: NR	Absolute speed: 103 mm/s mean glide Normalized speed: 0.515 BL/s Operating duration: NR Payload/ratio: 70 g block; separate 31 g camera	Test conditions: Water‐tank gliding and cargo tests Limitations: 70° is not a measured maximum; payload ratio requires whole‐robot operating mass
Jelly‐like muscle robot [98]	Size: 90 mm × 30 mm Mass: NR Actuation: Dielectric‐elastomer muscle Power source: Onboard battery Capacity/input: 500 mAh	Absolute speed: 9.1 mm/s Normalized speed: 0.3 BL/s Operating duration: 15.7 min Payload/ratio: NR	Test conditions: Underwater laboratory demonstration Limitations: repairability does not establish lifetime
Copebot [100]	Size: 200 mm Mass: 2 kg Actuation: Combustion‐driven expansion of soft membrane Power source: Preloaded O_2_/propane; onboard ignition and control electronics Capacity/input: O_2_:propane = 4.8:1; 16–26 mL examples for maneuvering	Absolute speed: 1.4 m/s Normalized speed: 7 BL/s Operating duration: NR Payload/ratio: NR	Test conditions: Laboratory tank: 1.2 × 1.2 × 2.4 m for maneuvering Limitations: 850 BL/s^2^ is the acceleration. Refilling autonomy and repeat‐jump endurance are not established by the cited interval
Surface‐slipping acoustic microrobot [180]	Size: 25 µm Mass: NR Actuation: Trapped‐bubble oscillation in a polymer shell Power source: External ultrasound; magnetic steering Capacity/input: 330 kHz	Absolute speed: 2.25 mm/s Normalized speed: 90 BL/s Operating duration: bubble retained ≥5 h under acoustic exposure Payload/ratio: NR	Test conditions: Liquid chamber near flat or curved solid boundaries Limitations/ gaps: Boundary‐dependent motion; bubble stability is not swimming endurance. Compliant element is not the shell
Tubular hydrophobic micromotor [202]	Size: 10 µm Mass: NR Actuation: Oscillating trapped bubble Power source: External ultrasound Capacity/input: NR	Absolute speed: 11 mm/s Normalized speed: 1100 BL/s Operating duration: NR Payload/ratio: NR	Test conditions: Liquid environments Limitations: Full text and supplement not verified. Tube deformation as a functional soft component is not established
Multiorifice acoustic microrobot [203]	Size: 10–100 µm diameter; fastest example ∼20 µm Mass: NR Actuation: Oscillating trapped bubble Power source: External ultrasound Capacity/input: 371 kHz for speed–voltage test	Absolute speed: 6 mm/s Normalized speed: 320 BL/s Operating duration: NR Payload/ratio: NR	Test conditions: PBS, blood serum, and glycerol‐water mixtures Limitations: Rounded absolute/normalized speeds are not exact conversions. Speed decreases in viscous media; deforming element is the trapped interface
Hovering acoustic microswimmer [204]	Size: 20 µm Mass: NR Actuation: Bubble streaming lifts the shell; acoustic radiation drives lateral motion Power source: External ultrasound Capacity/input: 250–450 kHz; peak speed near 370 kHz	Absolute speed: 350 mm/s Normalized speed: 17 500 BL/s Operating duration: NR Payload/ratio: NR	Test conditions: Near‐substrate motion in PBS–water solution Limitations: Highest speed is not sustained navigation; trajectories can curl. Rigid shell with deforming bubble interface
Lattice hydrogel microrobot [208]	Size: 100 µm × 50 µm × 20 µm Mass: NR Actuation: Photothermal lattice deformation Power source: External scanned laser Capacity/input: 780 nm; 60 mW and 10–14 Hz scanning	Absolute speed: 0.01515 mm/s Normalized speed: 0.14 BL/s, Operating duration: NR Payload/ratio: NR	Test conditions: Deionized water; locomotion on submerged glass Limitations: Optical access and precise scanning required; excessive heating causes floating or damage
LCG 70 swimmer [209]	Size: 16 mm × 3 mm × 0.1 mm Mass: NR Actuation: Light‐induced bending and buoyancy change of liquid crystal gel Power source: External illumination Capacity/input: below 1.8 W/cm^2^	Absolute speed: 4.9 mm/s Normalized speed: 0.3 BL/s Operating duration: NR Payload/ratio: NR	Test conditions: Salt‐water brine Limitations: Requires illumination of the middle of the body and adjusted fluid density; stroke speed is not whole‐cycle mean speed
Energy‐gradient surface robot [213]	Size: 45 mm Mass: 0.78 g Actuation: Released material induces interfacial and bulk‐liquid energy gradients Power source: Onboard energy‐releasing material; DBSA for peak‐speed test Capacity/input: NR	Absolute speed: Peak 250 mm/s Normalized speed: 5.5 BL/s Operating duration: NR Payload/ratio: NR	Test conditions: Water surface; Limitations: Not submerged swimming; speed decays and depends on medium and material release
IRONSperm [217]	Size: 60 µm Mass: NR Actuation: Magnetic bending of nanoparticle‐coated Power source: External rotating magnetic field Capacity/input: 2 mT	Absolute speed: 0.0108 mm/s Normalized speed: 0.18 BL/s Operating duration: 72 h drug‐release Payload/ratio: DOX‐HCl: 4.3 ± 0.2 pg per cell	Test conditions: In vitro liquid experiments; separate drug‐loading/release assays Limitations: Step‐out above ∼8 Hz; not powered by living sperm metabolism. Single‐cell ultrasound tracking and in vivo delivery not demonstrated
Biohybrid OstraBot [218]	Size: 30 mm Mass: NR Actuation: skeletal muscle drives compliant tendons and twin tails Power source: Muscle biochemical energy; external electric‐field stimulation Capacity/input: 0.50 V/mm, 3 Hz, 20 ms pulses at top speed	Absolute speed: 7.78 mm/s Normalized speed: 0.26 BL/s Operating duration: NR Payload/ratio: NR	Test conditions: In vitro buffer; submerged carbon electrodes provide stimulation Limitations: Hybrid PLA body, PDMS tendons and muscle; higher field (0.70 V/mm) reduces speed. Sound is a command, not propulsion power
Tissue‐engineered ray [219]	Size: 16.3 mm Mass: 10.18 ± 0.43 mg Actuation: Optogenetically activated cardiomyocytes bend elastomer/gold fins Power source: Glucose in physiological solution; external optical pacing Capacity/input: 470 nm; 1.5–2 Hz gives highest speeds	Absolute speed: 1.85 mm/s Normalized speed: 0.113 BL/s Operating duration: ≥80% speed retained for 6 days with daily tests Payload/ratio: NR	Test conditions: Tyrode's solution at 35°C–37°C Limitations: Requires viable tissue and controlled medium; culture lifetime and locomotion endurance are distinct

C. Aerial environments (Section 2.3)			
System and reference	Architecture and energy	Quantitative performance	Test conditions/limitations
RF‐powered flyer [125]	Size: Length 126 mm, height 60 mm Mass: 1.81 g Actuation: Piezoelectric flapping Power source: External RF transmitter; onboard receiver Capacity/input: 30 W transmitter; 750 mW received power	Absolute speed: 250 mm/s Normalized speed: 1.98 BL/s Operating duration: Short liftoff demonstration Payload/ratio: NR	Test conditions: Powered region 70–150 mm above transmitter Limitations: Transmitter power is not robot consumption; limited power‐transfer region
FlyJelly [128]	Size: Height 135 mm; wingspan 63 mm Mass: 270 mg Actuation: Electrostatic flapping; helium buoyancy Power source: Precharged onboard balloon capacitor Capacity/input: 19.457 mJ at 2,400 V; actuator power 0.3370 mW	Absolute speed: 96.7 mm/s Normalized speed: 0.72 BL/s Operating duration: >30 s untethered flight Payload/ratio: NR	Test conditions: Disconnected before takeoff; buoyancy‐assisted flight Limitations: Do not compare directly with heavier‐than‐air lift‐supported flight
DEA airship [129]	Size: Length 8000 mm Mass: NR Actuation: Dielectric‐elastomer undulatory tail; helium buoyancy Power source: Onboard batteries Capacity/input: Two 14.8 V, 2,100 mAh packs	Absolute speed: 450 mm/s Normalized speed: 0.056 BL/s Operating duration: NR Payload/ratio: NR	Test conditions: Mean speed measured over 10 m Limitations: 750 mm/s design prediction excluded
RGBlimp‐Q [130]	Size: NR Mass: 200.90 g Actuation: Moving‐mass continuum arm; helium gliding Power source: Onboard battery Capacity/input: 7.4 V, 150 mAh; 1.11 Wh nominal (C); 1.8 mWh/m reported	Absolute speed: 400 mm/s Normalized speed: NR Operating duration: 27 min over 605 m Payload/ratio: NR	Test conditions: Indoor tests; separate outdoor wind 0.5–1.5 m/s, gusts up to 3 m/s Limitations: Wind observations are not certified tolerance limits; author version must match cited publication
Light‐responsive artificial seed [134]	Size: Diameters 25 / 20 / 15 mm Mass: NR Actuation: Passive aerodynamics; light‐driven LCE shape change Power source: Ambient airflow and external light Capacity/input: NR	Absolute speed: Downward speeds 458 / 487 / 656 mm/s respectively Normalized speed: 18.32 / 24.35 / 43.73 BL/s respectively Operating duration: NR Payload/ratio: NR	Test conditions: Free‐descent tests Limitations: Passive descent is a separate category; suspended self‐oscillation is excluded from free‐flight evidence
Wind‐dispersed wireless sensor [220]	Size: 21.4 mm Mass: 30 mg Actuation: Passive aerodynamic dispersal Power source: Wind and gravity for dispersal; solar cells for electronics Capacity/input: NR	Absolute speed: 870 mm/s Normalized speed: 40.7 BL/s Operating duration: NR Payload/ratio: NR	Test conditions: Gentle to moderate outdoor breeze Limitations: Wind‐dependent transport; no active steering established
Dandelion microflier: descent [221]	Size: 20 mm Mass: 4 mg Actuation: Photothermal PI/LDPE bimorph opens pappus Power source: External light; gravity drives descent Capacity/input: 50 mW/cm^2^ lamp irradiation in open‐state descent test	Absolute speed: 450 mm/s Normalized speed: 22.5 BL/s Operating duration: NR Payload/ratio: 0–24 mg tested	Test conditions: Confined air; tracked height decreases from 200 to 34 mm Limitations: Limited control; no attitude feedback. Tested load is not an ascent payload capacity
LCN samara: free descent [222]	Size: 61 mm Mass: 33.1 mg Actuation: Photochemical deformation of azo‐LCN wing Power source: Gravity/wind; external UV/blue light adjusts wing Capacity/input: UV: 360 nm, 150 mW/cm^2^	Absolute speed: 1 m/s Normalized speed: 16.4 BL/s Operating duration: NR Payload/ratio: Tens of mg reported	Test conditions: Outdoor release: 14 m; wind ∼4 m/s; separate laboratory free‐fall tests Limitations: Passive descent, not powered flight. Thread‐guided wind‐tunnel height‐control results excluded from untethered comparison

D. Systems for in vivo applications (Section 2.4)			
System and reference	Architecture and energy	Quantitative performance	Test conditions/limitations
Hydrogel BAMs [145]	Size: 30 µm Mass: NR Actuation: Acoustic propulsion; magnetic guidance Power source: External ultrasound and magnetic field Capacity/input: NR	Absolute speed: PBS 2.043 ± 0.280 mm/s; whole blood 0.121 ± 0.019 mm/s Normalized speed: PBS 68.1 ± 9.3 BL/s; whole blood 4.033 ± 0.633 BL/s Operating duration: release assessed over 12 h Payload/ratio: 5‐FU 183.1 ng per 1000 BAMs	Test conditions: Biofluid tests; separate mouse‐bladder and tumour experiments Limitations: Biofluid speeds are not in vivo speeds; bubble retention over days is not functional endurance
Multilegged millirobot [151]	Size: 17 mm Mass: ∼ 45 mg Actuation: Magnetic deformation of compliant legs Power source: External magnetic field Capacity/input: NR	Absolute speed: 0.5 mm/s Normalized speed: 0.03 BL/s Operating duration: 50 s transport demonstration Payload/ratio: 91.4 mg tablet	Test conditions: Stomach model; separate dry high‐load test Limitations: >100× dry carrying result cannot be assigned to stomach transport
Micro‐clampbot [155]	Size: Length 883 µm Mass: NR Actuation: Acoustic propulsion and grasping Power source: External ultrasound Capacity/input: NR	Absolute speed: 0.106 mm/s Normalized speed: 0.120 BL/s Operating duration: 144 s route demonstration Payload/ratio: NR	Test conditions: In vitro PBS; 0.9 mm PDMS channel narrowing to 0.6 mm Limitations: Route average differs from reported motion speed; biological cargo is not in vivo validation
Ciliary‐band microrobot [157]	Size: 280 µm Mass: NR Actuation: Ultrasound‐excited compliant cilia Power source: External ultrasound Capacity/input: 68.8 kHz, 20 Vpp	Absolute speed: 2.6 mm/s Normalized speed: 9.3 BL/s Operating duration: NR Payload/ratio: NR	Test conditions: In vitro aqueous experiments Limitations: 10 mm/s tracer‐flow value is not robot speed; no demonstrated in vivo navigation
Peptide‐nanotube algae [160]	Size: 0.01 mm Mass: NR Actuation: Phototactic algal propulsion Power source: Cellular metabolism; external directional light Capacity/input: NR	Absolute speed: 0.0314 mm/s Normalized speed: 3.14 BL/s Operating duration: NR Payload/ratio: FITC encapsulation 90 ± 0.1% (SE)	Test conditions: In vitro motility; separate intratumoral mouse PTX treatment Limitations: Model FITC loading is not PTX dose; animal injection does not establish in vivo phototaxis
Amphibious Kresling origami millirobot [212]	Size: 6.8 mm Mass: NR. Actuation: Magnetic rolling, flipping, jumping, and spinning‐induced swimming; folding/unfolding for liquid delivery. Power source: External magnetic field. Capacity/input: ground locomotion: 10 mT, 4 Hz; peak swimming: 10 mT, 30 Hz.	Absolute speed: 81.2 mm/s Normalized speed: 11.9 BL/s Operating duration: NR. Payload/ratio: Solid‐cargo transport and liquid delivery demonstrated	Test conditions: Ground, water, air–water interface, and ex vivo pig stomach Limitations: External magnetic equipment required; swimming density adjusted close to water. Pumping uses a separate two‐magnetic‐plate configuration. Endurance, tracking error, and in vivo performance not established.

Table notes. Reference numbers follow the manuscript. BL/s denotes body lengths per second. NR indicates that the metric was not reported in the source material reviewed.

**FIGURE 6:**
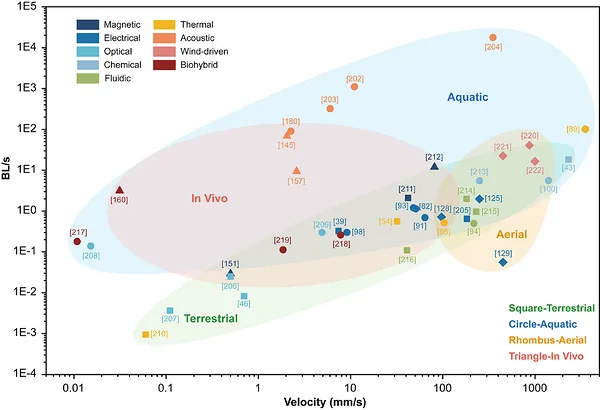
Locomotion performance of untethered soft robots across habitats and actuation modalities. Absolute speed (mm/s) is plotted against size‐normalized speed (body lengths per second, BL/s). Marker shape encodes operating habitat (square, terrestrial; circle, aquatic; diamond, aerial; triangle, in vivo); color encodes actuation modality. Shaded regions mark the approximate area occupied by each habitat and serve as visual guides, not performance bounds. Aerial values correspond to terminal descent speed under gravity rather than active propulsion.

Electrical actuation includes several distinct transduction mechanisms: electric‐field‐induced stress in dielectric elastomers, electromechanical coupling in piezoelectrics, thermally induced transformation after Joule heating in shape‐memory alloys, ion redistribution in ionic actuators, and electrically generated pressure in electrohydraulic systems. These mechanisms differ in voltage, bandwidth, deformation, and thermal requirements. Electrical input is readily modulated, but practical integration also depends on insulation, power conversion, and compatibility with the operating medium. On terrestrial substrates, electrical actuation is particularly effective because ground contact provides a stable reaction frame and the surrounding environment generally imposes fewer constraints on high‐voltage operation [35, 39, 40, 42, 54, 70, 162, 163]. In underwater environments, electrical actuation has been widely used for fin‐ and tail‐driven swimming, while recent plasticized electrohydraulic systems have extended operation to depths exceeding 3000 m [70, 71, 73, 74, 76, 90, 91, 93, 98, 99, 101, 106, 164]. In the air, piezoelectric and DEA flapping mechanisms provide most of the bandwidth and power density that have so far supported insect‐scale sustained flight [6, 16, 114, 117, 119, 123, 125, 127, 128, 129, 131, 165, 166, 167]. The main limitations of electrical actuation become pronounced in vivo, where high driving voltages raise safety concerns in biological tissues and onboard power sources are difficult to miniaturize below the millimeter scale. Electrical actuation is therefore largely absent from in vivo micro‐ and nanorobotic platforms, where it is replaced by magnetic and acoustic modalities.

Magnetic actuation uses field‐induced torque to orient or deform a magnetized body and magnetic‐field gradients to exert net translational forces. Rotation or deformation can also produce locomotion through contact with a substrate or interaction with a fluid. Because the robot can receive mechanical input without an electrical connection, this route is attractive for miniature systems in water and tissue; its accessible workspace nevertheless depends on the field source and the forces available at the required distance. These features make magnetic actuation particularly attractive for in vivo robots, ranging from millimeter‐scale gastric crawlers to submillimeter vascular micro‐fiberbots [141, 142, 150, 151, 152, 153, 154, 168, 169, 170, 171, 172, 173]. It is also widely used in terrestrial milli‐ and micro‐robots for crawling, rolling, and shape morphing [174, 175, 176]. In aquatic environments, magnetic actuation is effective at shallow depths but becomes less practical in the deep sea, where the increasing separation between the external field source and the robot weakens the field gradient available for propulsion [83, 177]. For aerial systems, magnetic actuation is rarely employed because available magnetic composites cannot generate sufficient lift against gravity at distances compatible with practical external field sources [178]. A cross‐cutting limitation is the need for external field‐generation hardware, such as permanent‐magnet arrays or Helmholtz coils, which often constrain the operational workspace. In situ magnetization reprogramming can partially alleviate this limitation by expanding the range of deformations achievable under a single uniform field. Nevertheless, workspace size and field‐source portability remain major bottlenecks for scaling magnetic actuation toward field deployment.

Acoustic actuation uses pressure oscillations to generate radiation forces, excite bubbles or compliant structures, and create streaming flows that produce propulsion or deformation. Its effectiveness depends on acoustic coupling, frequency, geometry, and the properties of the surrounding fluid. Bubble‐mediated swimmers exploit oscillation‐driven streaming in aquatic and biological fluids, whereas acoustic communication alone, as used in the remotely operated fish, is a control link rather than a propulsion mechanism [94, 179, 180, 181]. In vivo, focused ultrasound has demonstrated millimeter‐level spatial selectivity and Newton‐level force output via phase‐transition‐driven inflation, and frequency‐selective microbubble arrays have achieved programmable artificial‐muscle deformations with sub‐100 ms response [145, 182, 183, 184]. The main limitation of acoustic actuation is its reliance on an efficient coupling medium. In air, strong acoustic impedance mismatch, scattering, and attenuation greatly reduce energy transfer efficiency, making acoustic actuation far less practical for most terrestrial deployments than for submerged or in vivo environments. At great ocean depths, elevated hydrostatic pressure can shift bubble resonance, suppress volumetric oscillation, and reduce actuation efficiency.

Chemical actuation converts reaction energy into motion through mechanisms such as gas generation or rapid heating and expansion. Fluidic actuation instead describes the use of pressure and flow to deform chambers or exchange momentum with the environment; that pressure may be generated chemically or supplied by a pump or reservoir. These routes can therefore overlap, but their constraints differ: chemical systems require reactant management, while fluidic systems require an adequate pressure supply, flow control, and sealing under the ambient pressure. Among chemical actuators, combustion‐driven systems can achieve exceptionally high instantaneous power density, making them attractive for terrestrial jumping and impulsive underwater repositioning [43, 95, 100, 185]. However, the need for fuel storage, ignition components, and exhaust management limits miniaturization and restricts their use in enclosed or biologically sensitive environments. Fluidic actuation is effective on land, where compressed‐air reservoirs or onboard pumps can supply the working medium [57, 186]. Underwater operation imposes additional constraints, including reliable sealing against fluid ingress and the compressibility of gaseous working media under hydrostatic pressure. Pneumatic and hydraulic actuation nonetheless remain viable: continuum body undulation can be driven by onboard fluidic chambers in autonomous robotic fish [90], and pulsatile pneumatic chambers combined with soft‐chamber buckling have enabled scalable jet propulsion in untethered swimmers [164]. For aerial systems, the mass and volume of pneumatic supply systems are generally incompatible with the strict weight budgets of flying platforms [187]. Passive aerial systems exchange momentum with the surrounding air through drag, lift, autorotation, or aeroelastic deformation. Their motion may be driven by wind or by the conversion of gravitational potential energy during descent. Consequently, release height and airflow conditions are part of the energy and performance description, even when no onboard power is consumed [130, 132, 133, 135, 188].

Direct optical actuation produces deformation through photochemical changes or photothermal heating. Thermal actuation uses temperature‐dependent expansion, phase transformation, or changes in internal stress, whether heat is supplied optically, electrically, or by the environment. Optical access determines where energy can be deposited, while heat transfer constrains response and recovery. Photovoltaic powering is a separate route in which light supplies electricity to an actuator rather than directly deforming the robot. On land, light‐responsive materials such as liquid crystal elastomers and photothermal composites have enabled crawling, jumping, and adhesion switching [41, 46, 189, 190, 191]. Optical energy delivery supports aerial systems through both direct material actuation and photovoltaic powering, as illustrated by laser‐illuminated photovoltaics [118], onboard photovoltaic arrays under concentrated illumination [6], natural sunlight harvesting [126], and direct light‐induced shape change in LCE‐based microfliers [134]. By contrast, optical penetration is severely attenuated in turbid water and biological tissue, restricting direct photoactuation to surface or near‐surface operation in such media [77, 84]. Thermal actuation faces an analogous constraint: its response speed is governed by heat diffusion, which imposes an intrinsic trade‐off between actuation bandwidth and robot size, leaving thermally driven actuators generally slow at the centimeter scale but capable of faster cycling at the microscale, where thermal mass is small. On land, photothermal and Joule‐heated composites, including stacked‐graphene assemblies, shape‐memory polymer networks, and wirelessly powered bilayer structures, have enabled programmable crawling, gripping, and reconfigurable locomotion [192, 193, 194, 195]. In aquatic settings, thermal actuation has been used to drive shape‐memory swimming gaits, phase‐change buoyancy regulation, and bistability‐based directional propulsion [72, 96, 196].

Biohybrid actuators use cellular metabolism to supply muscle contraction or microorganism motility. Their compatibility with a habitat depends on both the mechanical task and the temperature, chemical conditions, and nutrient supply required to sustain the biological component. Responses to biochemical or optical cues can contribute to task execution, while externally commanded motion may require additional steering or feedback [147, 197, 198, 199, 200, 201].

Taken together, these modality‐specific constraints are not randomly distributed across habitats, but instead follow three recurring patterns. First, the environment acts as a selective filter for actuation strategies. Terrestrial settings impose relatively few constraints and therefore accommodate the broadest range of actuation methods, whereas aerial systems are largely centered on electrical actuation supplemented by passive aerodynamic structures, and in vivo systems mainly rely on magnetic, acoustic, and biohybrid modalities. Second, no single actuation modality is optimal across all environments. Each habitat favors modalities whose energy‐transmission pathways, such as electrical conduction, magnetic field penetration, acoustic propagation, optical irradiation, or chemical diffusion, are compatible with the local medium. Third, removing a physical tether changes the location and form of supporting infrastructure. An onboard pump or receiver adds mass, whereas external field generators can restrict range and workspace. Physical untethering therefore does not by itself establish independence from external equipment. Together, these patterns imply that untethered soft robots intended for complex or multi‐domain scenarios will increasingly depend on hybrid actuation architectures combining two or more complementary modalities, such as magnetic steering with acoustic force generation, or electrical locomotion with chemical energy release, to circumvent the limitations of any single approach.

Figure 6 compares the absolute and body‐length‐normalized speeds of selected untethered systems. These measures answer different questions: absolute speed describes the rate of displacement, whereas BL/s expresses that displacement relative to a defined body length. Neither measure alone establishes task effectiveness. Interpretation also requires the tested configuration, locomotion mode, environment, and whether the reported value represents peak or sustained motion. Descent speed in passive aerial systems is a separate measure of flight behavior and should not be interpreted as equivalent to propulsive speed.

Each modality occupies a characteristic region. Acoustic micro‐swimmers reach the highest BL/s, by up to three orders of magnitude, as a direct consequence of propelling bodies only tens of micrometers long, yet their absolute speeds remain modest [145, 157, 180, 202, 203, 204]. Electrical soft robots can achieve comparable absolute speeds, but their normalized speeds in body lengths per second usually fall within an intermediate range due to their centimeter‐scale dimensions [39, 82, 91, 93, 98, 125, 128, 129, 205]. Optical [46, 206, 207, 208, 209] and thermal [54, 89, 96, 210] actuators occupy the lower‐left region, indicating low speeds on both axes because their responses are bounded by light penetration depth and heat diffusion. Magnetic systems span a relatively broad region because magnetic fields can actuate robots across a wide range of length scales and can enable both precise control and relatively high locomotion speeds [151, 211, 212]. Chemical [43, 100, 213] and fluidic [94, 214, 215, 216] systems populate the high absolute‐speed band and supply the fastest locomotors in real terms. Biohybrid actuators cluster at low absolute speed, reflecting the limited power output of living‐cell motors [160, 217, 218, 219].

Habitat sets the accessible region in turn. Terrestrial systems bracket the absolute‐speed axis, reaching both the highest absolute speed and the lowest size‐normalized speed, and draw on the broadest set of actuation mechanisms among the non–in vivo habitats [39, 43, 46, 54, 94, 205, 206, 207, 210, 211, 214, 215, 216]. Aquatic systems span a broad range of absolute and normalized speeds in the selected dataset. Acoustic microswimmers illustrate how small body length can yield high BL/s despite modest absolute displacement, while surface propulsors demonstrate that high normalized speed is not exclusive to acoustic actuation [82, 89, 91, 93, 94, 96, 98, 100, 180, 202, 203, 204, 208, 209, 213, 217, 218, 219]. Peak values should be distinguished from sustained operating speeds when interpreting these examples. Aerial systems form a compact, high‐speed cluster set by passive descent under gravity rather than active propulsion [125, 128, 129, 220, 221, 222], and in vivo systems occupy a compact, low‐to‐moderate‐speed region served almost exclusively by magnetic and biohybrid actuation [145, 151, 157, 160, 212]. The performance plane therefore renders the environment‐actuation coupling quantitative: the region a system can reach is jointly constrained by the physics of its habitat and the mechanism of its actuator.

Flexibility and controllability provide additional, distinct dimensions of comparison. Here, flexibility denotes the functional deformation or reconfiguration that enables a robot to accommodate an environmental constraint; controllability denotes the demonstrated regulation of direction, orientation, trajectory, or locomotion mode. The latter is used in this operational sense, rather than as a formal claim of state‐space controllability. Relevant measures include passable constriction size relative to body dimensions, reversible changes in shape or gait, turning radius, tracking error, response time, and the source of feedback. A deformable body or an adjustable actuation signal alone does not establish these task‐level capabilities.

Representative systems illustrate why the two dimensions should be assessed separately. The light‐powered LCE robot deformed to pass through a channel approximately 25% lower than its initial body height [46], demonstrating geometric accommodation under a prescribed illumination sequence. Liang et al.’s untethered electrical crawler followed a designated light‐defined path using onboard photoresistors and a control circuit [39], providing evidence of sensor‐informed trajectory regulation. Ren et al.’s magnetic sheet changed locomotion mode to negotiate different fluid‐filled confinements [152], linking reconfiguration to externally commanded navigation. In the acoustically communicated robotic fish of Katzschmann et al., hydraulic body deformation and operator‐selected commands supported three‐dimensional maneuvering [94]. These examples establish different combinations of deformation, steering, and feedback under their respective test conditions; they are not interchangeable measures of overall control quality.

Actuation modality influences these capabilities without determining them by itself. External magnetic fields can command orientation and deformation but constrain the accessible workspace; acoustic actuation may require geometry, boundaries, or an additional steering mechanism to direct motion. Electrically driven systems can exploit integrated sensing and signal modulation, while light‐ and heat‐driven systems depend on stimulus access and response times. Impulsive chemical release places much of trajectory control before take‐off, whereas continuously regulated fluidic actuation permits adjustment during motion. Biohybrid responses to environmental cues likewise need to be distinguished from externally specified trajectory control. Comparisons should therefore report the demonstrated task, feedback arrangement, and operating conditions rather than assign a universal controllability ranking to actuator classes.

Performance comparisons are most informative within a specified task and operating regime. A speed advantage becomes relevant only if the system also meets the required load, duration, navigation, and environmental‐tolerance constraints. Conversely, a slower platform may be more suitable when accurate positioning, passage through a constriction, or sustained operation is the limiting requirement. Cross‐habitat comparisons should therefore identify the conditions under which a design is effective, while avoiding a single overall ranking of systems tested at different scales and under different conditions.

## 4 Outlook

The preceding sections highlight the substantial progress of untethered soft robots across diverse operating environments. Nevertheless, several cross‐cutting challenges must be addressed before these systems can transition from laboratory demonstrations to sustained deployment in the real world. We organize these challenges from material‐level concerns, to system‐level integration barriers, and ultimately to broader capability frontiers. Beyond identifying unresolved challenges, the following subsections extract practical design rules that link habitat‐specific constraints to materials, system architecture, actuation, and energy‐delivery strategies.

### 4.1 Material Longevity: Sustainability, Degradation, and Self‐Healing

Material selection must reconcile functional stability during the mission with recovery or degradation after use [19, 223]. Terrestrial and aerial systems encounter abrasion, ultraviolet exposure, thermal cycling, and repeated loading, motivating recyclable or reprocessable networks for repeated use and appropriately degradable materials for selected short‐lived applications [223, 224, 225]. Aquatic systems additionally require control of water uptake, hydrolysis, and biofouling; composites can separate load‐bearing and functional roles, provided that their interfaces remain stable during operation (Figure 7A) [19, 226]. For biomedical deployment, material lifetime, degradation products, and host response must be evaluated against the intended residence time and task [4, 227].

**FIGURE 7:**
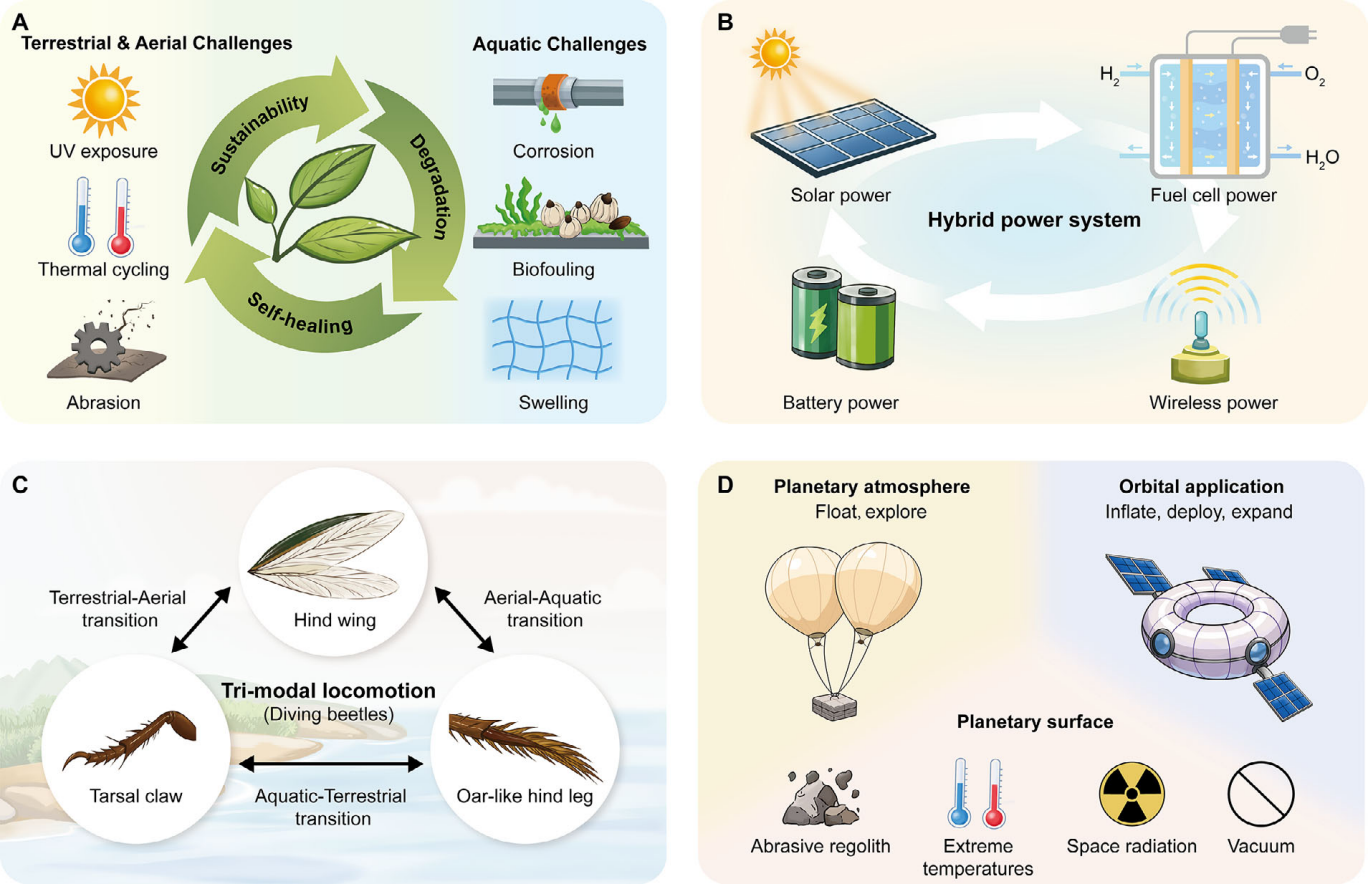
Cross‐cutting challenges and emerging directions for untethered soft robots, organized from material‐level constraints to broader capability frontiers. (A) Habitat‐specific material longevity. Adaptive soft materials must withstand ultraviolet (UV) exposure, thermal cycling, abrasion, corrosion, biofouling, and swelling across terrestrial, aerial, and aquatic environments, while simultaneously enabling sustainability, programmable degradation, and self‐healing. (B) Habitat‐matched onboard power. Hybrid power architectures that combine solar harvesting, batteries, fuel cells, and wireless power transfer are likely to outperform any monolithic energy source for sustained untethered deployment. (C) Multi‐environment locomotion. Diving‐beetle‐like designs, integrating wings, claws, and oar‐like appendages, exemplify the morphological reconfiguration required for transitions among land, water, and air within a single platform. (D) Extraterrestrial deployment. Soft robots offer compliance‐based impact tolerance and low launch mass for planetary atmospheric floating, orbital deployable structures, and surface operation under regolith abrasion, extreme temperatures, radiation, and vacuum.

An actionable selection rule is to define both the required operating lifetime and the intended end‐of‐life route before choosing the material. Recoverable platforms should be assessed for repair and reuse; a degradable design should retain the required function throughout the mission and degrade under the conditions it will actually encounter. Self‐healing should be evaluated by recovery of system function after representative damage, including leakage, electrical discontinuity, and interfacial failure, rather than by bulk‐material recovery alone [228, 229]. Where healing cannot restore a failed interface or embedded component, strain limitation, compartmentalization, replaceable modules, or damage‐triggered changes in operation are needed [230, 231, 232, 233].

### 4.2 Onboard Power and Energy Autonomy

Energy architecture must satisfy the mission's total energy demand and peak power demand while remaining compatible with the robot's mass, deformation, and operating environment (Figure 7B) [2]. Batteries provide portable storage but introduce packaging and mechanical‐integration constraints [6, 234]. Fuel cells require fuel storage and balance‐of‐system components [235]. Photovoltaic supply can reduce dependence on stored energy when adequate illumination is available, as illustrated by laser‐ and sunlight‐powered aerial platforms [118, 126]. Wireless power transfer offers an alternative where onboard storage is restrictive, but its usefulness depends on coupling, range, alignment, and the surrounding medium [236, 237, 238]. Direct magnetic or acoustic actuation should also be distinguished from power transfer to an onboard electrical receiver.

Selection should begin with a mission power budget that includes sensing, computation, communication, and power conversion. Onboard storage is appropriate when that complete budget fits within the available mass and volume; external supply requires sufficient delivered power throughout the intended workspace. For jumping and other transient behaviors, the source and any intermediate storage must meet peak demand as well as the integrated energy requirement. A hybrid architecture is justified when harvesting or local buffering improves mission completion or endurance enough to offset its added mass and complexity. Its evaluation should report the external infrastructure and supply conditions alongside the robot's onboard resources, so that untethered operation is not conflated with energy independence.

### 4.3 Multi‐Environment Transition

Multi‐environment locomotion can be achieved by adapting the motion of a shared structure or by reconfiguring between structures suited to different media [239, 240]. Land–water robots illustrate the first approach through appendages that generate substrate reaction forces on land and hydrodynamic forces in water [241, 242, 243]. Transitions involving air impose additional requirements for aerodynamic force generation and mass reduction. Air–water crossing also introduces interface forces and transient loading [244, 245], whereas controlled descent or gliding can extend the reach of a terrestrial platform without requiring sustained powered flight [246, 247, 248, 249, 250, 251]. The combination of aquatic, terrestrial, and aerial functions exemplified by diving beetles remains a demanding integration objective for soft robots (Figure 7C) [252, 253].

The design decision is whether a common structure can satisfy the required forces, motion range, and stability in each medium after all onboard subsystems are included. If it can, shared actuation can reduce hardware duplication. If it cannot, reconfiguration or separate propulsion structures should be considered, with their mass and switching cost included in the comparison. Transition performance must be assessed separately from performance within each habitat: a system that moves effectively in air and water may still fail at the interface. Repeated transition trials should therefore report completion rate, energy cost, recovery time, and whether external intervention is required.

### 4.4 Extraterrestrial Applications

Extraterrestrial deployment requires adaptation to the specific combination of gravity, atmosphere, terrain, and exposure conditions at the destination (Figure 7D) [254, 255, 256]. Surface hopping offers a potential route in reduced gravity, but its usefulness depends on control of launch direction, landing, and subsequent recovery [257, 258]. Planetary atmospheric vehicles require adequate buoyant or aerodynamic support under local conditions [259]. In orbit, inflatable and deployable structures can reduce stowed volume, although deployment reliability and the required deployed geometry remain important constraints [260, 261, 262, 263, 264, 265, 266]. Materials, seals, and electronics must also tolerate the thermal, radiation, vacuum, or abrasive conditions relevant to the selected mission [267, 268, 269, 270].

These requirements should be translated into design tests before architecture selection. A hopper should be evaluated for controlled landing and repeatable relaunch, an atmospheric platform for weight support and maneuvering under the local density, and a deployable structure for deployment repeatability and shape retention. Environmental testing should assess operation during exposure, as well as survival and performance afterward. Architectures whose essential energy supply, cooling, or control functions depend on unavailable infrastructure should be rejected or redesigned at this stage.

### 4.5 Perception Control and Task Autonomy

Task autonomy should be assessed through the connection between observation, decision‐making, and corrective action. Reports should identify the observed state variables, the location of sensing and computation, and the actions the robot or its external controller can select without human intervention. For deformable bodies, this connection may change with loading, material drift, damage, or the surrounding medium. Calibration and feedback should therefore be evaluated under the conditions of the intended task, rather than only for an unloaded actuator.

A practical design rule is to identify the failure that would prevent mission completion and select a sensing and control architecture capable of detecting and addressing it in time. Examples include contact loss during climbing, depth drift during swimming, attitude divergence during flight, and loss of localization during biomedical navigation. The resulting decision may be to correct the trajectory, switch gait, pause, or terminate the task. Repeated trials should report completion rate, tracking error, intervention frequency, and behavior after loss of power, communication, or imaging. Physical untethering, energy autonomy, and task autonomy should be reported as separate system attributes.

## 5 Conclusion

Untethered soft robots are best understood as systems whose architecture is set, not chosen freely, by the physics of the habitat in which they operate. The operating environment is therefore not merely a deployment context, but a primary determinant of material selection, structural design, actuation strategy, and achievable function. Across the four habitats surveyed, the dominant constraints differ qualitatively: terrain negotiability and energy efficiency on land; capillary equilibrium, hydrodynamic drag, and pressure tolerance in depth‐stratified aquatic environments; lift‐to‐weight optimization under severe mass and power limits in air; and miniaturization, biocompatibility, and wireless control through opaque tissues in vivo. Actuation, structural, and material choices that succeed in one habitat often do not transfer directly to another. This helps explain why progress in untethered soft robotics has accumulated as habitat‐specific solutions rather than a single convergent design paradigm. The same logic applies to actuation: no single modality is optimal across all habitats. Each environment filters the available actuation landscape according to the compatibility of energy transmission, force generation, and material chemistry with local physical and biological conditions. Recognizing this environment‐actuation coupling as a primary design constraint, rather than an isolated engineering choice, reframes how viable systems should be conceived and evaluated. Translating habitat‐bound demonstrations into sustained deployment will require coordinated advances in material durability, onboard energy autonomy, multi‐environment transition, and robust closed‐loop operation. Achieving these advances will depend on whether habitat‐specific design rules can be recombined into systems that satisfy the constraints of multiple habitats simultaneously.

## Author Contributions

**Siyang Li**: conceptualization, software, data curation, formal analysis, validation, visualization, project administration, Writing – original draft, investigation, methodology. **Zhikun Miao**: conceptualization, investigation, writing – original draft, validation, visualization, software, formal analysis, project administration, data curation, methodology. **Tuck‐Whye Wong**: conceptualization, supervision, formal analysis, validation, investigation, project administration, writing – original draft, visualization, methodology. **Tiefeng Li**: conceptualization, investigation, writing – original draft, validation, formal analysis, project administration, supervision, methodology.

## Conflicts of Interest

The authors declare no conflicts of interest

## Data Availability

The data that support the findings of this study are available from the corresponding author upon reasonable request.
